# Comparative Analysis of the Chloroplast Genome for *Aconitum* Species: Genome Structure and Phylogenetic Relationships

**DOI:** 10.3389/fgene.2022.878182

**Published:** 2022-05-31

**Authors:** Conglong Xia, Manjiong Wang, Yunhui Guan, Jian Li

**Affiliations:** ^1^ State Key Laboratory of Bioreactor Engineering, Shanghai Key Laboratory of New Drug Design, East China University of Science and Technology, Shanghai, China; ^2^ College of Pharmacy, Dali University, Dali, China

**Keywords:** *Aconitum*, medicinal plants, chloroplast genome, comparative analysis, phylogenomics

## Abstract

*Aconitum* is an important medicinal group of the Ranunculaceae family and has been used as conventional medicine in Bai, Yi, and other ethnic groups of China. There are about 350 *Aconitum* species globally and about 170 species in China. It is challenging to identify the species in morphology, and the lack of molecular biology information hinders the identification and rational utilization of the germplasm of this genus. Therefore, it is necessary to increase the molecular data of *Aconitum* species. This paper acquired the complete chloroplast (CP) genome sequence of ten medicinal plants of *Aconitum* species from Yunnan by Illumina paired-end (PE) sequencing technology and compared it with other species in the same family and genus. These CP genomes exhibited typical circular quadripartite structure, and their sizes ranged from 155,475 (*A. stylosum*) to 155,921 bp (*A. vilmoinianum*), including a large single-copy region (LSC), a small single-copy region (SSC), and two inverted repeat regions (IRs). Their gene content, order, and GC content (38.1%) were similar. Moreover, their number of genes ranged from 129 (*A. vilmoinianum*) to 132 (*A*. *ramulosum*), including 83 to 85 protein-coding genes (PCGs), 37 tRNA genes (tRNAs), eight rRNA genes (rRNAs), and two pseudogenes. In addition, we performed repeated sequence analysis, genomic structure, and comparative analysis using 42 *Aconitum* chloroplast genomes, including ten *Aconitum* chloroplast genomes and other sequenced *Aconitum* species. A total of 48–79 simple sequence repeats (SSRs) and 17 to 77 long repeat sequences were identified. IR regions showed higher variability than the SSC region and LSC region. Seven mutational hotspots were screened out, including *trnK*-*UUU*-*trnQ*-*UGG*, *psbD*, *ndhJ*-*ndhK*, *clpP*, *psbH*-*petB*, *ycf1*, and *trnA*-*UGC*-*trnI*-*GAU*, respectively. The phylogenetic trees of ten *Aconitum* species and other *Aconitum* species revealed that the complete CP genome was beneficial in determining the complex phylogenetic relationships among *Aconitum* species. This study provides a potential molecular marker and genomic resource for phylogeny and species identification of *Aconitum* species and an important reference and basis for Ranunculaceae species identification and phylogeny.

## Introduction

*Aconitum* belonging to the Ranunculaceae comprises approximately 350 species mainly distributed in Asia, followed by Europe and North America. There are about 170 species of *Aconitum* in China. The Qinghai-Tibet Plateau and its adjacent areas are its most extensive distribution region, and the northern provinces are its second-largest distribution region (Li and Kadota, 2001).

*Aconitum carmichaelii* Debx. has been used in China for more than 2,000 years. It was first recorded in Shennong Bencao Jing (Wu and Sun, 1936). Seventy-six species of *Aconitum* plants were used in folk medicine through textual research, mainly for treating heart failure, rheumatism, joint pain, falling injury, stroke, paralysis, sore boils and poison, and wind and cold syndrome (Yang, 2012). However, there were differences in application, such as *A*. *episcopale* could detoxify, has anti-alcoholic properties, and detoxifies opium (Li et al., 1995); *A*. *brachypodum* was used for fractures, sprain, rheumatism, fall injury (Yang et al., 2016); and *A*. *alboviolaceum* was used for hypertension joint pain (Lu, 1995). It is important to note that many of the species of *Aconitum* plants are also reported to be toxic. The application process is highly susceptible to endangering human life safety due to misused species (He et al., 2010). The variety of *Aconitum* species and the diversity of the natural environment in the distribution area make its morphological variation extremely complex. Thus, it is difficult to accurately identify the *Aconitum* species based on their morphological characteristics. As for molecular identification, previous studies have demonstrated that barcoding sequences (ITS2, *psbA*-*trnH*) are also not ideal for accurate identification of *Aconitum* spp. (Zhu et al., 2020). Therefore, finding an accurate identification marker for this genus is necessary. Chloroplasts are independent organelles in the plant cells, have a complete set of the genome, which is relatively conserved in the genetic composition structure and contains more abundant mutation sites. These structural features, which allow chloroplast genomes to occupy a vital position in plant species’ discrimination and evolutionary study, have been widely used as super barcodes for species identification and phylogenetic studies (Sugiura, 1992; Yurina and Odintsova, 1998; Moore Michael et al., 2007).

In this study, we assembled and analyzed the complete chloroplast genomes sequence of ten medicinal plants of *Aconitum* species from Yunnan using Illumina PE sequencing and reported the CP genomes of ten medicinal plants of *Aconitum* species, including *Aconitum stapfianum* Hand.-Mazz., *A. episcopale* Leveille, *A. ramulosum* W.T.Wang, *A. vilmoinianum* Kom., *A. nagarum* Stapf., *A. ouvrardianum* Hand.-Mazz., *A. delavayi* Franch., *A. duclouxii* Levl., *A. stylosum* Stapf., and *A. weixiense* W.T.Wang. We performed a general characteristic analysis of chloroplast genomes for the ten *Aconitum* species sequenced in this study. In addition, we performed repeated sequence analysis, genome structure, comparative analysis, and phylogenetic analysis of the ten *Aconitum* species in this study and 32 chloroplast genomes of *Aconitum* submitted to the GeneBank database. We aim to expand our understanding of the genome divergence of *Aconitum*, provide insights into the phylogenetic relationships of *Aconitum* species, and identify potential DNA barcodes for identifying *Aconitum* species. These results will provide a theoretical basis for molecular phylogenetic and evolutionary studies at the species level of *Aconitum.*

## Materials and Methods

### Plant Materials

**FIGURE 1:**
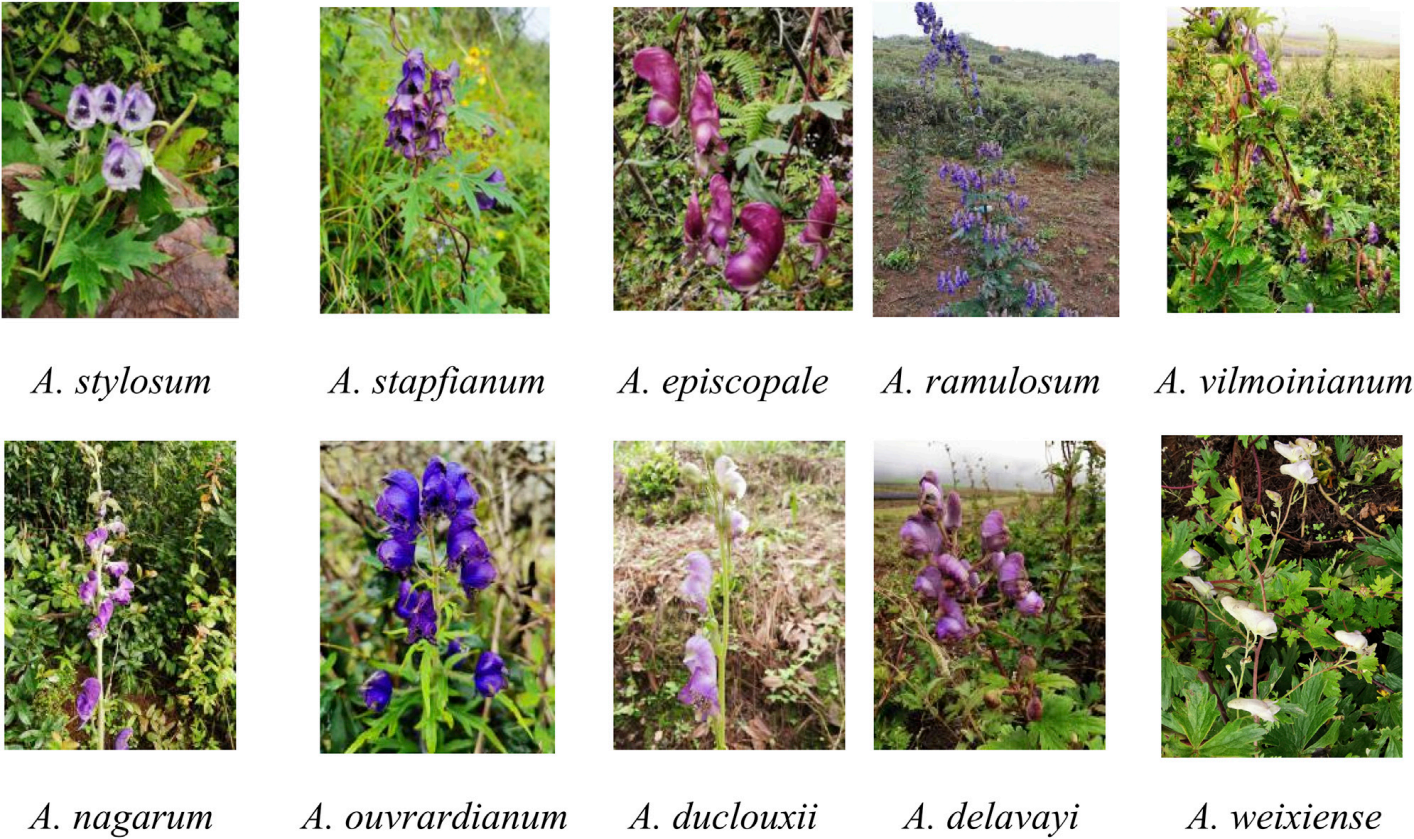
Pictures of original plants of ten *Aconitum* spp.

Plant materials from ten *Aconitum* spp. were collected from the Weixi County, Lijiang County, and Zhongdian County, Yunnan Province, in September 2020 (Table 1; Figure 1). Detailed information about the materials used and sequences obtained in the study is provided in Table 1. Fresh leaf material without lesions was collected and stored dry with discoloured silica gel. Professor Conglong Xia of Dali University identified the species according to the morphological characteristics recorded in the Flora of China. The voucher specimens were deposited in the Plant and Medicinal Herbology, College of Pharmacy, Dali University. The voucher specimens number was recorded as WT001∼WT0010.

**TABLE 1:** Information on the collection of ten *Aconitum* spp.

Sample ID	Species	Collection dates	Collection locations	Altitude	Longitude and Latitude	Preservation method
WT001	*A. vilmoinianum*	2020.09	Zhongdian county	3315.91	27°90′38″ N 99°63′82″ E	Refrigerator (−20°C)
WT002	*A. stylosum*	2020.09	Deqing county	4285.73	27°90′38″ N 99°63′82″ E	Refrigerator (−20°C)
WT003	*A. episcopale*	2020.09	Weixi county	2752.50	26°85′99″ N 99°73′16″ E	Refrigerator (−20°C)
WT004	*A. stapfianum*	2020.09	Lijiang county	3633.98	27°04′77″ N 100°19′34″ E	Refrigerator (−20°C)
WT005	*A. weixiense*	2020.09	Heqing county	3109.36	26°48′01″ N 100°06′02″ E	Refrigerator (−20°C)
WT006	*A. nagarum*	2020.09	Lushui county	3130.40	25°51′26″ N 99°01′22″ E	Refrigerator (−20°C)
WT007	*A. duclouxii*	2020.09	Bingchuan county	2533.86	25°97′50″ N 100°68′26″ E	Refrigerator (−20°C)
WT008	*A. ouvrardianum*	2020.09	Deqing county	4285.73	28°33′97″ N 99°07′51″ E	Refrigerator (−20°C)
WT009	*A. delavayi*	2020.09	Heqing county	3109.36	26°48′01″ N 100°06′02″ E	Refrigerator (−20°C)
WT0010	*A. ramulosum*	2020.09	Heqing county	3109.36	26°48′01″ N 100°06′02″ E	Refrigerator (−20°C)

### DNA Extraction and Illumina Sequencing

Total genomic DNA was extracted from clean leaves from samples frozen at −80°C using the E.Z.N.A® Plant DNA kit (OMEGA, Beijing). Shanghai Origingene Co., Ltd. did the work. The DNA quality was assessed by electrophoresis in a 1% (w/v) agarose gel. The OD_260_/_280_ values ranged from 1.8 to 2.0, and >2 μg of DNA was equally pooled from individuals of the ten species to generate shotgun libraries. DNA samples were randomly sheared, incubated with fragmentation buffer, and broken into 300–500 bp fragments in an M220 focused ultrasonicator (Covaris, Woburn, MA, United States). The A & B connectors at both ends of the DNA fragment were connected, the segments were screened, and the self-connecting segments of the connector were removed. Fragment screening by electrophoresis in agarose gel keeps the fragment with A connector at one end, and B connector at the other end; the subsequent additions of NaOH to denatures produces single-stranded DNA fragments. This DNA was further paired-end sequenced using the Illumina Novaseq 2500 (Shanghai Origingene Co., Ltd., China) platform.

### Genome Quality and Control and De Novo Assembly

Raw image data obtained by Illumina sequencing was converted into FASTQ format sequencing data files by Base Calling (Paul, 2015). The FASTQ data file used FastQC v0.11.4 (www.bioinformatics.babraham.ac.uk/bugzilla/) for quality control. The sequenced reads were spliced with several iterations using NOVOPlasty (https://github.com/ndierckx/NOVOPlasty) stitching software (Dierckxsens et al., 2017). The seed sequence required for the assembly was MN556604.1 (complete chloroplast genome of *Delphinium grandiflorum*), and the optimal assembly result was obtained.

### Genome Annotation and Submission

PGA software (https://github.com/quxiajian/PGA) was used for gene annotation (Qu et al., 2019), and manual corrections were made for all sample annotation results. The annotated chloroplast genome GB file was submitted to the online tool (https://chlorobox.mpimp-golm.mpg.de/OGDraw.html) to generate a physical map (Lohse et al., 2007). Finally, these TBL files were generated to submit the sequences to NCBI. The complete and correct CP genome sequences of the ten *Aconitum* species were deposited in the GenBank database (https://www.ncbi.nlm.nih.gov/genbank/) of NCBI, and accession numbers are OM289057.1 (*A. ouvrardianum*), OM289058.1 (*A. delavayi*), OM289059.1 (*A. ramulosum*), OM328065.1 (*A. vilmoinianum*), OM328066.1 (*A. episcopale*), OM328067.1 (*A. stapfianum*), OM328068.1 (*A. nagarum*), OM328069.1 (*A. weixiense*), OM328070.1 (*A. duclouxii*), and OM328071.1 (*A. stylosum*), respectively.

### Codon Usage Analysis

The CodonW software (University of Texas, Houston, TX, United States) was used to investigate the distribution of codons based on the relative synonymous codon usage (RSCU) ratio and the effective number of codon usage (ENC) to analyze the codon usage preference (Sharp et al., 1986).

### Repeat Sequence Analysis

The FASTA files of whole chloroplast genome sequences of 42 *Aconitum* species were sorted out. Simple sequence repeats (SSRs) were detected using the MISA software (http://pgrc.ipk-gatersleben.de/misa/) (Beier et al., 2017). The minimum values for the number of repeats of mono -, di -, tri -, tetra -, penta -, and hexanucleotide repeats were set to 10, 5, 4, 3, 3, and 3 respectively. The sizes and locations of repeat sequences in the CP genomes of the 42 *Aconitum* spp. were identified using REPuter (http://bibiserv.techfak.uni-bielefeld.de/reputer/) (Kurtz et al., 2001) with the parameters set to a similarity percentage of scattered repeat copies ≥90%, a minimal repeat size of 30 bp, the hamming distance of 3 and maximum computed repeats of 5,000.

### Genome Structure and Comparative Genome Analysis

The mVISTA online program (https://genome.lbl.gov/vista/mvista/submit.shtml) (Frazer et al., 2004) was used in Shuffle-LAGAN model to compare 42 *Aconitum* species CP genome using *A*. *vilmorinianum* CP genome as a reference. The IRscope (https://irscope.shinyapps.io/irapp/) online program (Amiryousefi et al., 2018) was used to obtain IR regions comparative analysis of the chloroplast genome.

### Sliding Window Analysis

The DnaSP software was used for sliding window analysis (Rozas et al., 2017), the nucleotide diversity values (Pi) were calculated, and interspecific high variation sequences (hotspots) were screened according to the analysis results. The size of the windows length was set to 600 bp, and the step size was set to 200 bp.

### Phylogenetic Analysis

To determine the phylogenetic positions of the ten *Aconitum* species within Ranunculaceae, we analyzed the CP genomes of 44 species, encompassing 34 additional taxa within this lineage. The CP genome sequences of 34 species were downloaded from Genebank database. The complete CP genome sequences and PCGs were used to reconstruct the *Aconitum* species phylogenetic tree. The chloroplast genome sequences were aligned using MAFFT online tool (Katoh and Standley, 2013). We used the CP genomes of *Delphinium anthriscifolium* Hance (MK253461.1) and *Delphinium grandiflorum* L. (NC_049872.1) as outgroups. The MEGA X software (Kumar et al., 2018) was used to construct phylogenetic trees employing 44 CP genomes sequences based on neighbour-joining (NJ) with 1,000 bootstrap replicates, and the model was Kimura 2-parameter. The IQ-tree software (Nguyen et al., 2015) was used to construct phylogenetic trees employing 44 CP genomes sequences based on maximum likelihood (ML) with 1,000 bootstrap replicates.

## Results

### Chloroplast Genomes of Ten *Aconitum* spp.

**FIGURE 2:**
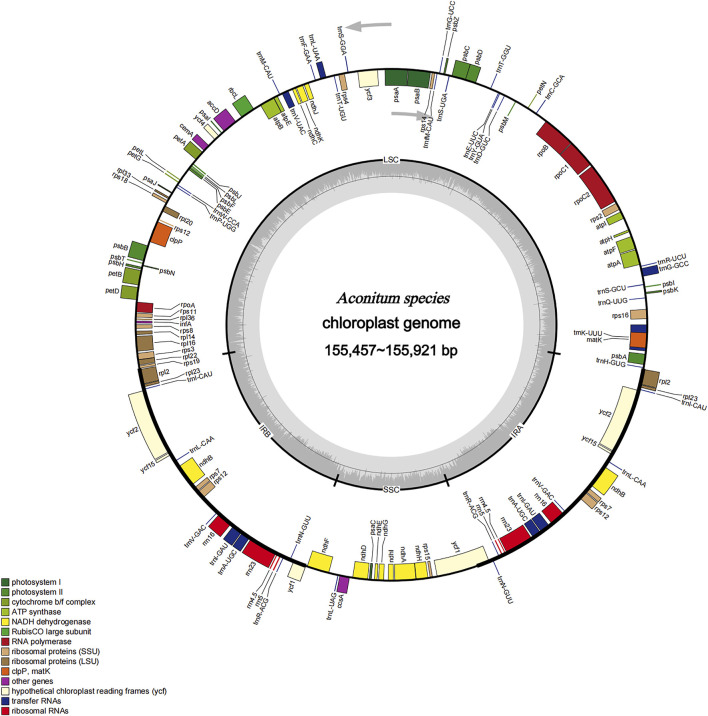
Gene map of the complete chloroplast genomes of *Aconitum* species. Genes inside the circle are transcribed clockwise, and those on the outside are transcribed counter-clockwise. Genes belonging to different functional groups have been colour-coded. The darker grey area in the inner circle corresponds to GC content, whereas the lighter grey corresponds to AT content.

The genome sequences assembled using the reads obtained from the Illumina sequencing platform ranged from 155,475 bp for *A. stylosum* to 155,921 bp for *A*. *vilmorinianum* (Table 2). The genome exhibited a typical cyclic tetramer structure, including the SSC and LSC regions separated by two IR regions (Figure 2). The sequence length of LSC regions ranged from 86,098 bp for *A*. *stylosum* to 86,524 bp for *A*. *vilmorinianum*, the sequence length of SSC regions ranged from 16,903 bp for *A*. *stylosum* to 16,983 bp for *A. nagarum*, and the sequence length of IRs regions ranged from 52,218 bp for *A*. *duclouxii* to 52,476 bp for *A. ouvrardianum* (Table 2).

**TABLE 2:** Basic characteristics of complete chloroplast genomes in ten *Aconitum* plants.

Species names	*A. vilmorinianum*	*A. stylosum*	*A. episcopale*	*A. stapfianum*	*A. weixiense*	*A. nagarum*	*A. duclouxii*	*A. ouvrardianum*	*A. delavayi*	*A. ramulosum*
Total length (bp)	155,921	155,475	155,853	155,858	155,872	155,732	155,479	155,799	155,733	155,841
LSC length (bp)	86,524	86,098	86,443	86,449	86,493	86,313	86,318	86,420	86,362	86,470
SSC length (bp)	16,925	16,929	16,938	16,937	16,921	16,983	16,943	16,903	16,913	16,913
IR length (bp)	52,472	52,448	52,472	52,472	52,458	52,436	52,218	52,476	52,458	52,458
Coding (bp)	79,014	78,906	78,996	78,996	78,978	78,984	78,891	78,324	78,258	78,366
Non-coding (bp)	76,907	76,569	76,857	76,862	76,894	76,748	76,588	77,475	77,475	77,475
Total number of genes	129	129	129	129	129	129	129	132	132	132
Total number of unique genes	111	111	111	111	111	111	111	114	114	114
protein-coding genes (duplicated)	83 (7)	83 (7)	83 (7)	83 (7)	83 (7)	83 (7)	83 (7)	85 (7)	85 (7)	85 (7)
tRNA gene (duplicated)	37 (7)	37 (7)	37 (7)	37 (7)	37 (7)	37 (7)	37 (7)	37 (7)	37 (7)	37 (7)
rRNA gene (duplicated)	8 (4)	8 (4)	8 (4)	8 (4)	8 (4)	8 (4)	8 (4)	8 (4)	8 (4)	8 (4)
Pseudogenes	1	1	1	1	1	1	1	2	2	2
GC content (%)	38.1	38.1	38.1	38.1	38.1	38.1	38.1	38.1	38.1	38.1
GC content of LSC (%)	36.2	36.2	36.2	36.2	36.2	36.2	36.2	36.2	36.2	36.2
GC content of IR (%)	43.0	43.0	43.0	43.0	43.0	43.0	43.0	43.0	43.0	43.0
GC content of SSC (%)	32.6	32.6	32.6	32.6	32.6	32.6	32.6	32.6	32.6	32.6
A (bp)	47,737	47,598	47,630	47,712	47,728	47,674	47,585	47,840	47,827	47,839
C (bp)	29,836	29,781	30,123	29,833	29,824	29,833	29,803	30,197	30,170	30,212
G (bp)	29,517	29,466	29,058	29,515	29,524	29,513	29,481	29,141	29,138	29,144
T (bp)	48,825	48,624	48,391	48,798	48,796	48,712	48,610	48,621	48,598	48,646

The chloroplast genomes of ten *Aconitum* species can be divided into the coding and non-coding regions; the coding region sequence lengths ranged from 79,014 to 78,996 bp, and the non-coding region sequence lengths ranged from 76,569 to 77,475 bp. The ten *Aconitum* chloroplast genome sequences all encoded 129–132 genes (including IR region duplicates), and if 18 duplicates in the IR regions were excluded, a total of 111–114 genes were encoded, including 83 to 85 PCGs (seven duplicated genes), 37 tRNAs (seven duplicated genes), eight rRNAs (four duplicated genes). It is noteworthy that two pseudogenes (*ycf1* and *rps19*) were found in *A*. *ouvrardianum*, *A*. *delavayi*, and *A*. *ramulosum*, whereas only the pseudogene *ycf1* was found in the remaining species. Moreover, the GC contents of the ten *Aconitum* CP genomes were 38.1%, and the IR regions (43%) had higher GC contents than the single-copy regions (LSC: 36.2% and SSC: 32.6%). Furthermore, we also analyzed the length and frequency of four bases that included adenine (A), thymine (T), guanine (G), and cytosine (C) in the chloroplast genome of ten *Aconitum* species. The results showed that the lengths of adenine ranged from 47,585 to 47,840 bp, the lengths of cytosine ranged from 29,781 to 30,212 bp, and the lengths of guanine ranged from 29,058 to 29,524 bp, and the lengths of thymine ranged from 48,391 to 48,825 bp, respectively. A and T were used significantly more frequently than G and C.

### Base Composition Analysis of Genome

The base composition of the entire coding region and different position codons in the chloroplast genomes of the ten *Aconitum* species were analyzed (Table 3). The coding region length ranged from 63,372 bp for *A*. *duclouxii* to 79,014 bp for *A*. *delavayi*. The total AT content in the coding region was 60.6–61.7%, and the total GC content was 38.3–39.4%. The length of the first, second, and third codons ranged from 21,124 bp to 26,338 bp, and GC contents ranged were 45.9–47.3% (first codon), 38.5–39.4% (second codon), and 30.7–31.5% (third codon), respectively. Moreover, the third codon site has AT preference.

**TABLE 3:** Base composition of coding regions.

	Protein coding					Frist position codon					Second position codon					Third position codon				
	T(U)%	C%	A%	G%	Total	T(U)%	C%	A%	G%	Total	T(U)%	C%	A%	G%	Total	T(U)%	C%	A%	G%	Total
*A. vilmorinianum*	31.1	17.8	30.5	20.5	79,014	23.5	18.9	30.6	27.0	26,338	32.2	20.5	29.4	18.0	26,338	37.7	14.2	31.6	16.5	26,338
*A. stylosum*	31.2	17.9	30.5	20.5	78,906	23.5	18.9	30.6	27.0	26,302	32.3	20.5	29.3	18.0	26,302	37.7	14.2	31.6	16.5	26,302
*A. episcopale*	31.1	17.9	30.5	20.5	78,456	23.5	18.9	30.6	27.1	26,152	32.2	20.5	29.3	18.0	26,152	37.7	14.2	31.6	16.5	26,152
*A. stapfianum*	31.1	17.8	30.5	20.5	78,996	23.5	18.9	30.6	27.0	26,332	32.2	20.5	29.3	18.0	26,332	37.7	14.2	31.6	16.5	26,332
*A. weixiense*	31.1	17.8	30.5	20.5	78,996	23.5	18.9	30.6	27.0	26,332	32.2	20.5	29.3	18.0	26,332	37.7	14.2	31.6	16.5	26,332
*A. nagarum*	31.1	17.8	30.5	20.5	78,996	23.5	18.9	30.6	27.0	26,332	32.2	20.5	29.3	18.0	26,332	37.7	14.2	31.6	16.5	26,332
*A. duclouxii*	30.6	18.4	30.0	21.0	63,372	22.8	19.5	29.9	27.8	21,124	31.5	21.0	29.1	18.4	21,124	37.5	14.7	31.0	16.8	21,124
*A. ouvrardianum*	30.5	18.3	30.1	21.0	63,543	22.7	19.4	30.0	27.9	21,181	31.4	20.9	29.2	18.4	21,181	37.5	14.7	31.1	16.8	21,181
*A. delavayi*	31.1	17.8	30.5	20.5	79,014	23.5	18.9	30.6	27.0	26,338	32.2	20.5	29.4	18.0	26,338	37.7	14.2	31.6	16.5	26,338
*A. ramulosum*	31.2	17.8	30.5	20.5	78,978	23.5	18.9	30.6	27.0	26,326	32.2	20.5	29.3	18.0	26,326	37.7	14.2	31.6	16.5	26,326

Furthermore, we also analyzed the base composition in different regions of the ten *Aconitum* species chloroplast genomes (Supplementary Table S1). The AT content of SSC was the highest, followed by the LSC region and IRs region. The AT content of the protein-coding gene region was the highest, followed by the tRNAs region and rRNAs region. The results indicated that the AT content of the chloroplast genome was higher than the GC content and also demonstrated the codon preference for using bases A and T(U).

### Genome Function and Classification

Chloroplast genomes of ten *Aconitum* species were annotated, and their genes were functionally classified (Table 4). The number of chloroplast genome coding genes in the ten *Aconitum* species ranged from 129 to 132, including 83–84 protein-coding genes (PCGs), 37 tRNA genes, and eight rRNA genes. The first group of genes involved in transcription and translation consists of 69–70 genes, including small subunit of ribosome genes (*rps2*, *rps3*, *rps4*, *rps7* × 2, *rps8*, *rps11*, *rps12* × 2, *rps14*, *rps15*, *rps16*, *rps18*, and *rps19*), ten large subunit of ribosome genes (*rpl2* × 2, *rpl14*, *rpl16*, *rpl20*, *rpl22*, *rpl23* × 2, *rpl33*, and *rpl36*), one transcription initiation factor genes (*infA*), four RNA polymerase genes (*rpoA*, *rpoB*, *rpoC1*, *rpoC2*), eight rRNA genes (*rrn16* × 2, *rrn23* × 2, *rrn4.5* × 2, *and rrn5* × 2), and 37 tRNA genes (*trnA-UGC* × 2, *trnC-GCA*, *trnD-GUC*, *trnE-UUC*, *trnF-GAA*, *trnfM-CAU*, *trnG-GCC*, *trnG-UCC*, *trnH-GUG*, *trnI-CAU* × 2, *trnI-GAU* × 2, *trnK-UUU*, *trnL-CAA* × 2, *trnL-UAA*, *trnL-UAG*, *trnM-CAU*, *trnN-GUU* × 2, *trnP-UGG*, *trnQ-UUG*, *trnR-ACG* × 2, *trnR-UCU*, *trnS-GCU*, *trnS-GGA*, *trnS-UGA*, *trnT-GGU*, *trnT-UGU*, *trnV-GAC* × 2, *trnV-UAC*, *trnW-CCA*, and *trnY-GUA*). The second group of 45 genes involved in the photosynthesis, including 20 Photosystem I and Photosystem II genes (*psaA*, *psaB*, *psaC*, *psaI*, *psaJ*, *psbA*, *psbB*, *psbC*, *psbD*, *psbE*, *psbF*, *psbH*, *psbI*, *psbJ*, *psbK*, *psbL*, *psbM*, *psbN*, *psbT*, and *psbZ*), six Cytochrome b/f complex genes (*petA*, *petB*, *petD*, *petG*, *petL*, *petN*), six ATP synthase genes (*atpA*, *atpB*, *atpE*, *atpF*, *atpH*, *atpI*), one ATP-dependent protease subunit gene (*clpP*), one RubiscoCO large subunit gene (*rbcL*), and 12 NADH oxidoreductase genes (*ndhA*, *ndhB* × 2, *ndhC*, *ndhD*, *ndhE*, *ndhF*, *ndhG*, *ndhH*, *ndhI*, *ndhJ*, *ndhK*). The third group of four genes involved in the biosynthesis of amino acids, fatty acids, etc., included one Maturase gene (*matK*), one Envelop membrane protein gene (*cemA*), one Subunit Acetyl-CoA-Carboxylate gene (*accD*), and one c-type cytochrome synthesis gene (*ccsA*); another group of unknown function genes has 6–8. Among the ten *Aconitum* species, *A*. *ouvrardianum*, *A*. *delavayi*, and *A*. *ramulosum* contained *ycf15* and *rps16* genes, and the other seven species did not have *ycf15* and *rps16* genes.

**TABLE 4:** List of genes in the chloroplast genome of ten *Aconitum* species.

Category	Group genes	Name of genes
Transcription and translation	Large subunit of ribosome (LSU)	rpl2* (x 2), rpl14, rpl16*, rpl20, rpl22, rpl23 (x 2), *rpl33, rpl36*
	Small subunit of ribosome (SSU)	*rps2*, *rps3*, *rps4*, *rps7* (x 2), *rps8*, *rps11*, *rps1*2** (x 2), *rps14*, *rps15*, *rps16*, *rps18*, *rps19* ^ψ^
	RNA polymerase	*rpoA*, *rpoB*, rpoC1*, *rpoC2*
	Translational initiation factor	*InfA*
	rRNA genes	*rrn16* (x 2), *rrn23* (x 2), *rrn4.5* (x 2), *rrn5* (x 2)
	tRNA genes	trnA-UGC* (x 2), *trnC-GCA*, *trnD-GUC*, *trnE-UUC*, *trnF-GAA*, *trnfM-CAU*, trnG-GCC*, *trnG-UC*C, *trnH-GUG*, *trnI-CAU* (x 2), *trnI-GAU* (x 2)*, trnK-UUU*, *trnL-CAA* (x 2), *trnL-UAA*, *trnL-UAG*, *trnM-CA*U, *trnN-GUU*(x 2), *trnP-UGG*, *trnQ-UUG*, *trnR-ACG* (x 2), *trnR-UCU*, *trnS-GCU*, *trnS-GGA*, *trnS-UGA*, *trnT-GGU*, *trnT-UGU*, trnV-GAC* (x 2), *trnV-UAC*, *trnW-CCA*,*trnY-GUA*
Photosynthesis	Photosystem I	*psaA*, *psaB*, *psaC*, *psaI*, *psaJ*
	Photosystem II	*psbA*, *psbB*, *psbC*, *psbD*, *psbE*, *psbF*, *psbH*, *psbI*, *psbJ*, *psbK*, *psbL*, *psbM*, *psbN*, *psbT*, *psbZ*
	NADH oxidoreductase	ndhA*, ndhB* (x 2), *ndhC*, *ndhD*, *ndhE*, *ndhF*, *ndh*G, *ndhH*, *ndhI*, *ndhJ*, *ndhK*
	Cytochrome b6/f complex	*petA*, petB*, petD*, *petG*, *petL*, *petN*
	ATP synthase	*atpA*, *atpB*, *atpE*, atpF*, *atpH*, *atpI*
	RubiscoCO large subunit	*RbcL*
	ATP-dependent protease subunit gene	clpP**
Other genes	Maturase	*MatK*
	Envelop membrane protein	*CemA*
	Subunit Acetyl- CoA-Carboxylate	*AccD*
	c-type cytochrome synthesis gene	*CcsA*
Unknown	Conserved Open reading frames	*ycf1* ^ψ^ (x 2), *ycf2* (x 2), ycf3**, *ycf4*, *ycf15* (x 2)

“×2” indicated duplication of the gene in the IR, region; “*” indicated that the gene contains an intron; “**” indicated that the gene contains two introns; “ψ” indicated the gene as a pseudogene.

In eukaryotic and semi-prokaryotic systems, gene expression occurs in the cytoplasm and organelles of the nucleus, respectively. Introns play a vital role in the regulation of gene expression. Previous studies have proved that introns can improve the expression level of extraneous genes in eukaryotic genomes (Cui, 2020). A total of 17 genes contained introns in the ten *Aconitum* CP genomes, *clpP*, *ycf3*, and *rps12* genes contained two introns and three exons, whereas the other 14 genes all contained one intron and two exons (Supplementary Table S2). In addition, the introns and exons contained by the genes of ten *Aconitum* were essentially the same or had minor differences in length, as shown by the results in Supplementary Table S2.

### Codon Usage Analysis

**FIGURE 3:**
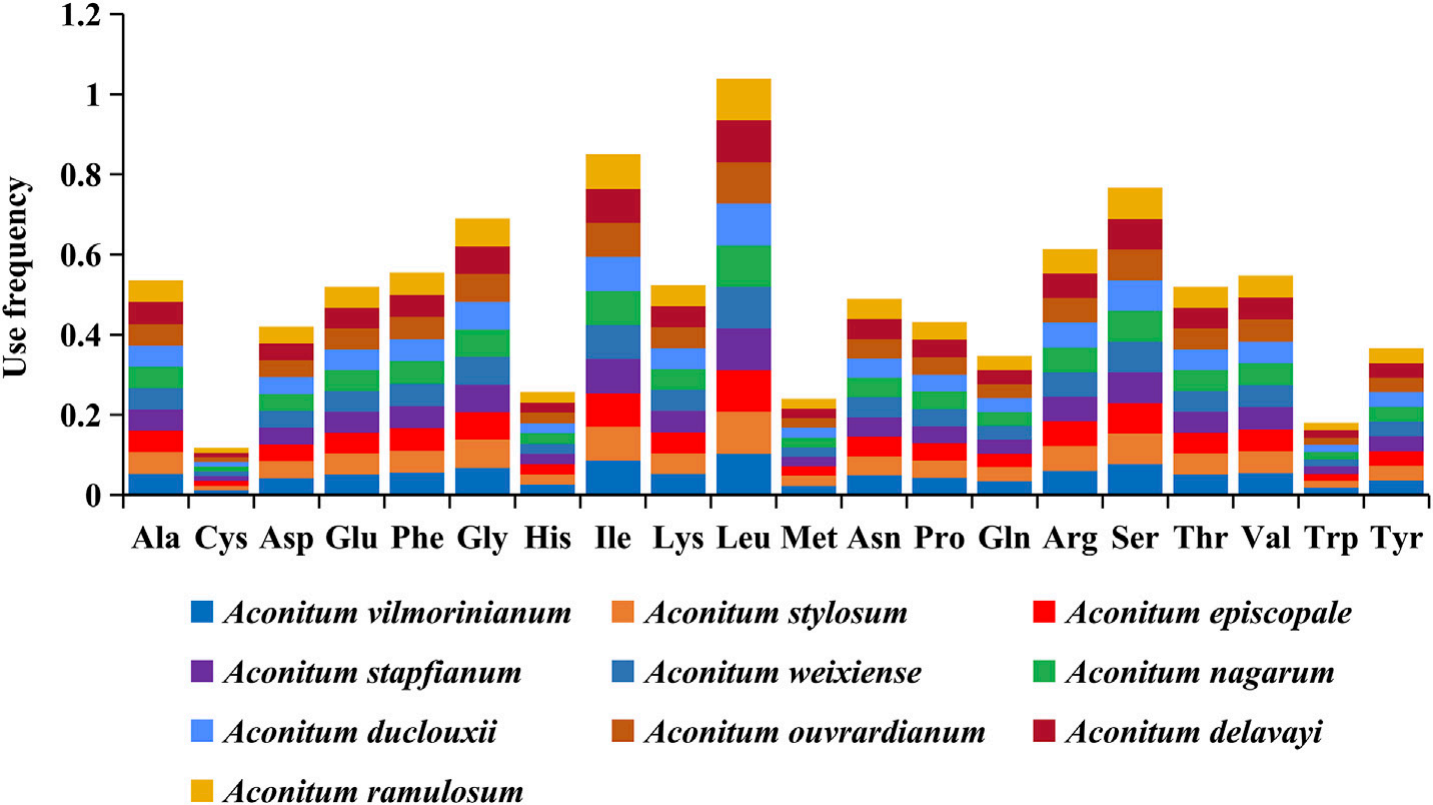
Frequency of amino acids encoded in the coding region by codons of chloroplast genes from ten *Aconitum* species.

The codon usage and codon recognition patterns of the ten *Aconitum* CP genomes are shown in Figure 3 and Supplementary Table S3. The CP protein-coding genes of these ten species contained 61 codons encoding 20 amino acids. Leucine (10.4%–10.5%) was the most frequent, and cysteine (1.18%–1.19%) was the least, which was consistent with the results of amino acids coding of chloroplast genomes in most plants (Somaratne et al., 2020; Wang Z. et al., 2021). The statistical results of the frequency of codon usage in the chloroplasts of the ten *Aconitum* species showed that the coding regions (CDS) comprised 26,086 codons in *A*. *ouvrardianum* to 26,338 codons in *A*. *vilmorinianum* (Supplementary Table S5). Among amino acids encoded by the codon, Leu was the most frequent amino acid, encoding 2,701 times in *A*. *ouvrardianum* to 2,732 times in *A*. *stylosum* and *A*. *nagarum*; The least frequently coded amino acid was Cys, only encoding 302 times in *A*. *ouvrardianum*, *A*. *ramulosum*, and *A*. *delavayi* to 307 times in *A*. *vilmorinianum*. The results are consistent with those in Figure 3.

**FIGURE 4:**
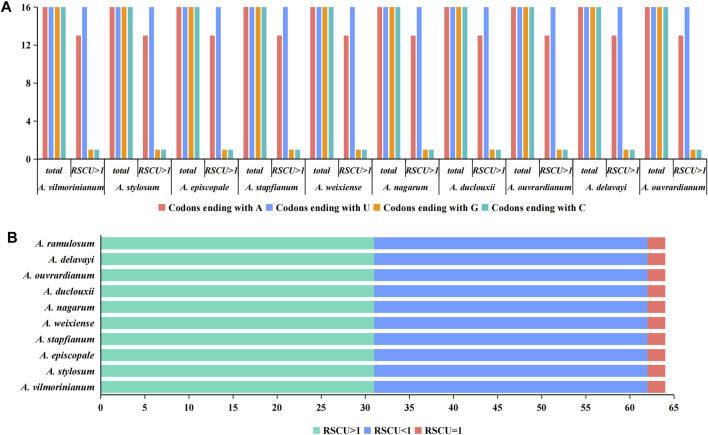
Analysis of codon bias in the chloroplast genome of ten *Aconitum* species. **(A)** Analysis of terminal bases at codons in chloroplast genomes; **(B)** RSCU value analysis of codons in chloroplast genomes.

Furthermore, we analyzed the relative synonymous codon usage (RSCU) and codon base composition of protein-coding gene sequences (CDS) of ten *Aconitum* species. There were 64 codons in CDS sequences of ten *Aconitum* species genes, among which 61 codons encoded 20 amino acids and the other three codons were stop codons (Figure 4B). An RSCU value <1.00 indicates that the codon usage frequency is lower than expected, whereas an RSCU value >1.00 indicates that the codon usage frequency is higher than expected (Sharp et al., 1986). In this study, the RSCU values of 31 codons were greater than 1, RSCU values of 31 codons were less than 1, and RSCU values of two codons were 1 (Figure 4B). Among the 64 codons, there were 16 codons, each ending with A, U, G, and C. Meanwhile, among the codons with RSCU value >1, 13 codons ended in A, 16 codons ended in U, one codon ended in G, and one codon ended in C (Figure 4A). These results indicated that the chloroplast genome codon of *Aconitum* species prefers to end in A/U.

### SSRs in the Chloroplast Genomes of the 42 *Aconitum* spp.

**FIGURE 5:**
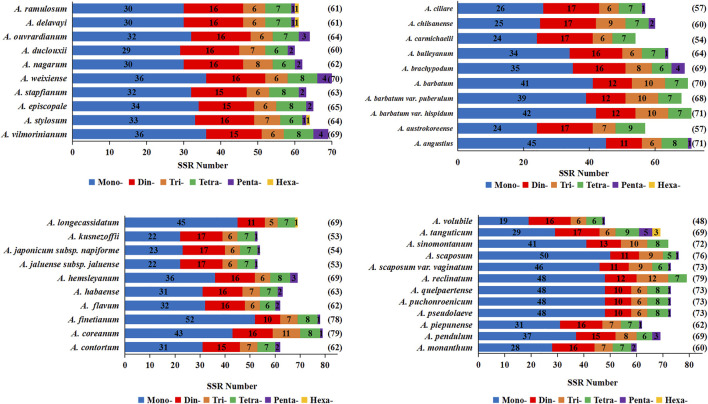
Analysis of simple sequence repeat (SSR) in the chloroplast genomes of 42 *Aconitum* species.

SSRs are widely present in CP genomes, consisting of 1-6-nucleotide repeat units, and are valuable molecular markers of high variation within the same species (Jiao et al., 2012). In order to explore the distribution and differences of SSRs among *Aconitum* species, we detected SSRs in the chloroplast genomes of 42 *Aconitum* species using MISA. Ten of them were the species sequenced in this study, and the sequences of the remaining 32 species were downloaded from NCBI. This study analyzed the number, type, and regional distribution of SSRs in 42 *Aconitum* chloroplast genomes (Figure 5). The total SSR loci in chloroplast genomes of 42 *Aconitum* species ranged from 48 in *A. volubile* to 79 in *A. coreanum* and *A*. *reclinatum.*

**FIGURE 6:**
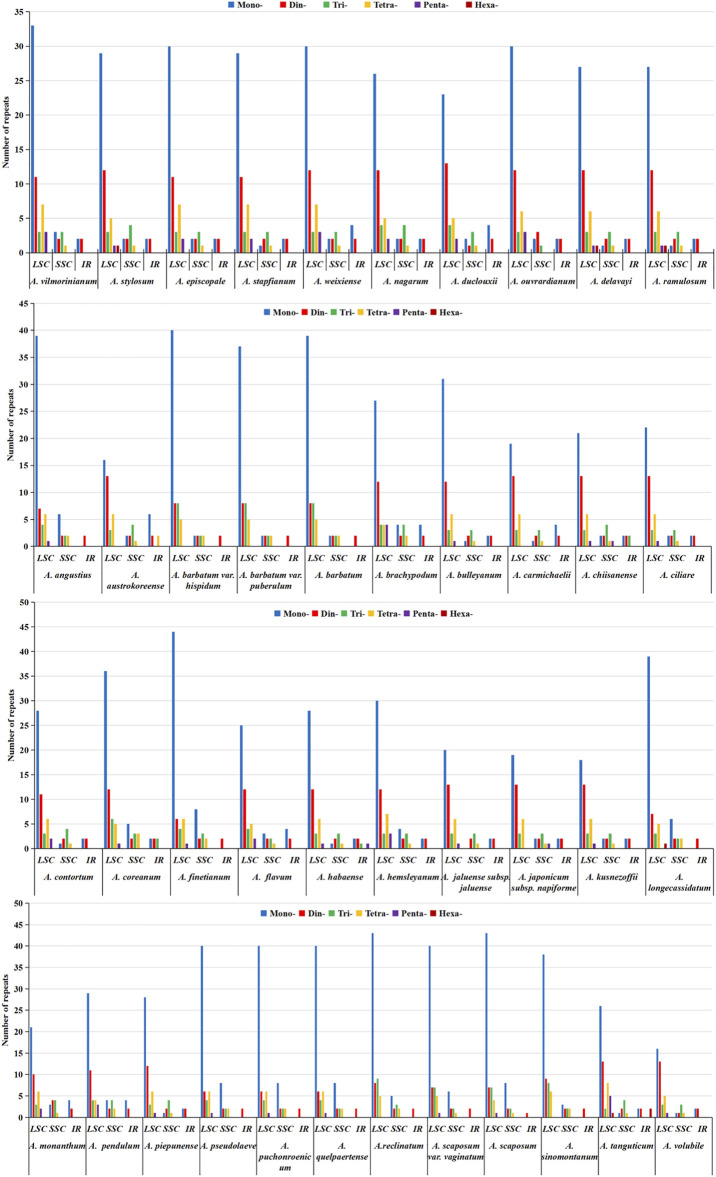
Distribution types and numbers of SSRs in LSC, SSC, IR regions in the chloroplast genomes of 42 *Aconitum* spp.

Furthermore, we analyzed SSRs loci in different regions of the chloroplast genome (LSC/SSC/IRs). The results showed that the distribution of SSRs significantly varied in different regions (Figure 6). The number of SSRs loci in the LSC region ranged from 38 to 65, accounting for 66.67%–85.92% of the total number of SSRs; SSC regions ranged from six to fifteen, accounting for 9.38%–20.00%; Two IR regions ranged from one to ten, accounting for 1.32%–17.54%. Most of the SSR sequences in the chloroplast genomes of 42 *Aconitum* plants were composed of mononucleotide and dinucleotide repeat units. The number of mononucleotide repeats ranged from 19 in *A. volubile* to 52 in *A. finetianum*. These were followed by dinucleotide (10–17), trinucleotide (6–12), tetranucleotide (6–9), pentanucleotide (1–5), and hexanucleotide (1–3).

We analyzed the repeat units of different types of repeat sequences in ten *Aconitum* species and found that A/T (96.67%–100% of the total SSRs of each species) and AT/TA (100%) dominated the repeat sequence types of mononucleotide and dinucleotide SSRs, respectively. The repeated trinucleotide SSRs were mainly composed of A/T base combinations (AAT/ATT, 71.43%–85.71%) (Supplementary Table S5). These results were consistent with previous reports that CP SSRs usually consist of short poly-A or poly-T repeats and rarely contain tandem G or C repeats in many plants (Kuang et al., 2011).

### Long Repeat Analysis

**FIGURE 7:**
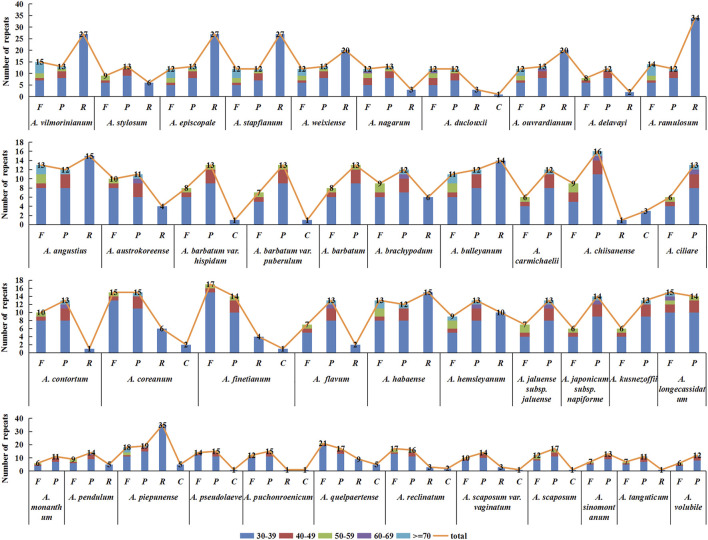
Type and number of long repeat sequences distributed in the chloroplast genomes of 42 *Aconitum* species.

In this study, the analysis results of long repeats in chloroplast genome sequences of 42 *Aconitum* species showed 1,467 long repeats in all species, and each species had 17–77 long repeats, respectively (Figure 7). Among them, *A*. *piepunense* has the most significant number of long repeats, including 18 forward, 19 palindromic, 35 reverse repeats, and five complement repeats; *A*. *monanthum* is the least, including six forward and 11 palindromic repeats. Among the 42 *Aconitum* species, *A*. *duclouxii*, *A*. *barbatum* var*. hispidum*, *A*. *barbatum* var. *puberulum*, *A*. *chiisanense*, *A*. *finetianum*, *A*. *coreanum*, *A*. *piepunense*, *A*. *pseudolaeve*, *A*. *puchonroenicum*, *A*. *quelpaertense*, *A*. *reclinatum*, *A*. *scaposum* var. *vaginatum*, and *A*. *scaposum* which contained 30–39 bp complement repeat sequence.

Of the 42 species, the length range of the repeats was divided into five types (30–39, 40–49, 50–59, 60–69, ≥70 bp), with the highest number of repeats of 30–39 bp in length. All *Aconitum* species contained 30–39 bp repeat sequences, accounting for 41.18%–85.71% of the total repeat sequences. Among them, 30–39 bp long repeats in *A*. *piepunense*, and *A*. *monanthum* are the most and least abundant, respectively.

The 60–69 bp repeat sequences were the least. Only in *A. nagarum*, *A. duclouxii*, *A. ouvrardianum*, *A. austrokoreense*, *A. brachypodum*, *A. chiisanense*, *A. ciliare*, *A. contortum*, *A. flavum*, *A. hemsleyanum*, *A. jaluense* subsp*. jaluense*, *A. japonicum subsp. napiforme*, *A. longecassidatum*, *A. pendulum* containing repeats of 60–69 bp in length, accounting for 3.57% , 3.70%, 2.22%, 4.00%, 3.70%, 3.45%, 5.26%, 4.17%, 4.55%, 3.13%, 5.00%, 5.00%, 3.45%, and 3.57% of the total repeat sequence.

### IR Boundary Variation Analysis

**FIGURE 8:**
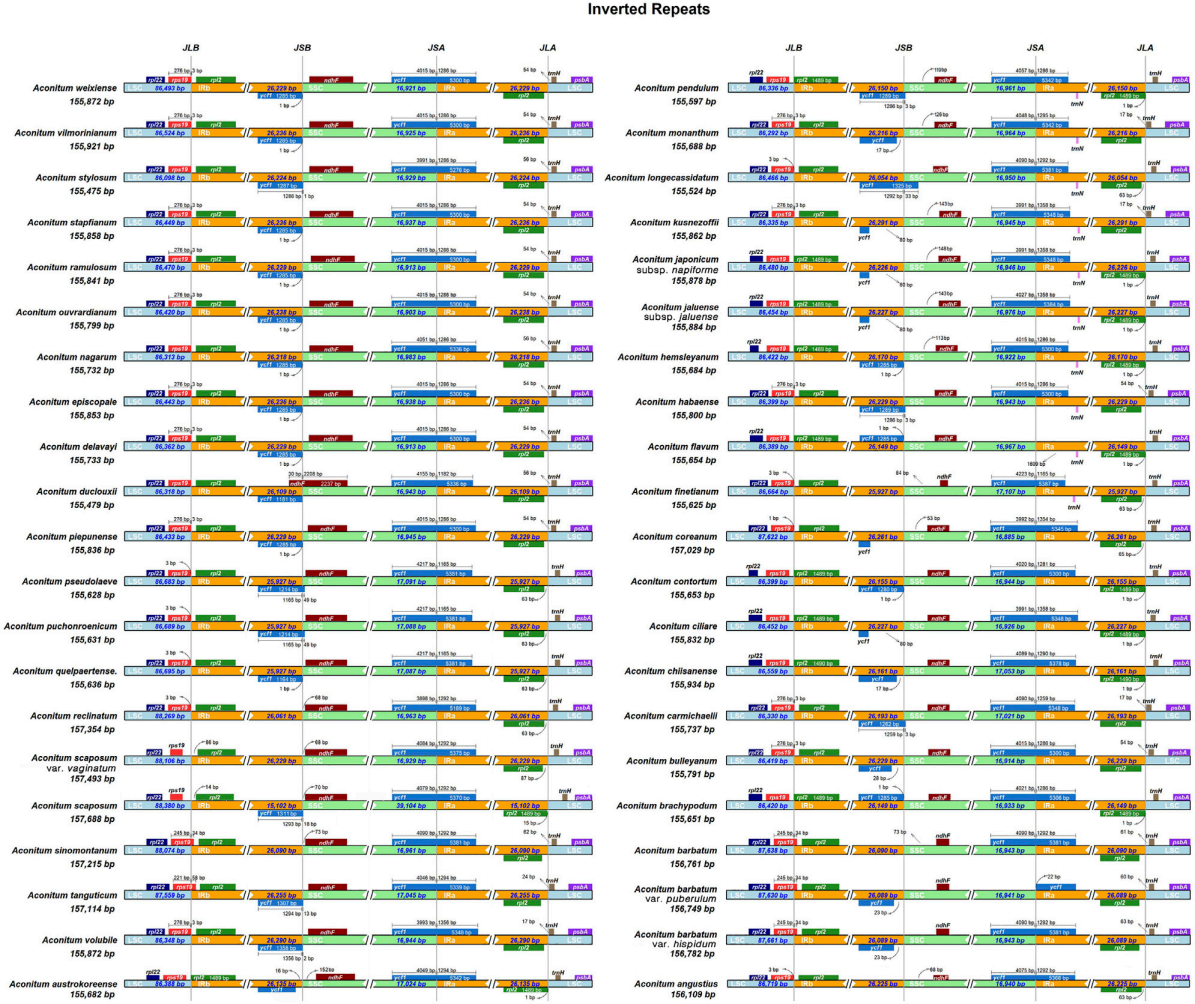
Comparison of LSC, SSC, and IR region borders among CP genomes of 42 *Aconitum* species.

During the adaptive evolution of plant species, the expansion and contraction of the IR boundary of the chloroplast genome resulted in different levels of sequence replication at the four boundaries (LSC/SSC/IRa/IRb) (Hu and Zhang, 2021). Analysis of IR boundaries of chloroplast genome sequences of 42 *Aconitum* species showed that the four boundaries, the structure of the chloroplast genome, and the connection between IR regions were very conserved; LSC/IR and SSC/IR boundary distribution genes included *rps19*, *rpl22*, *rpl2*, *ycf1*, *ndhF*, *trnH,* and *psbA* (Figure 8). The *rps19* gene in the chloroplast genomes of 42 *Aconitum* species showed various degrees of contraction and expansion at the LSC/IRb boundary. And then, the *rps19* gene in chloroplast genomes of most *Aconitum* species exhibited a 3 bp protrusion in the IRb region. As the *ycf1* gene straddles SSC/IRa boundary, a pseudogene ψ*ycf1* was generated in the IRb region. No *ycf1* pseudogene was found in *A*. *reclinatum*, *A. scaposum* var. *vaginatum*, *A. sinomontanum*, *A*. *finetianum*, *A*. *barbatum*, and *A. angustius* at the IRb region.

The length of the *ndhF* gene in *A*. *duclouxii* was 2,237 bp, extending across the SSC/IRa boundary to 30 bp in the IRa region, and the other *Aconitum* species all had *ndhF* gene of different lengths in the SSC region. These results indicated that some variation occurred in *A*. *duclouxii*. The *rpl22*, *trnH*, and *psbA* genes in the chloroplast genomes of the 42 *Aconitum* species were all located in the LSC region, the *rpl2* gene was all located in the IRa region, and these genes were essentially unchanged in length across species, which can be used as a common feature of *Aconitum* chloroplast genomes for species identification.

### Genomic Variation Analysis

**FIGURE 9:**
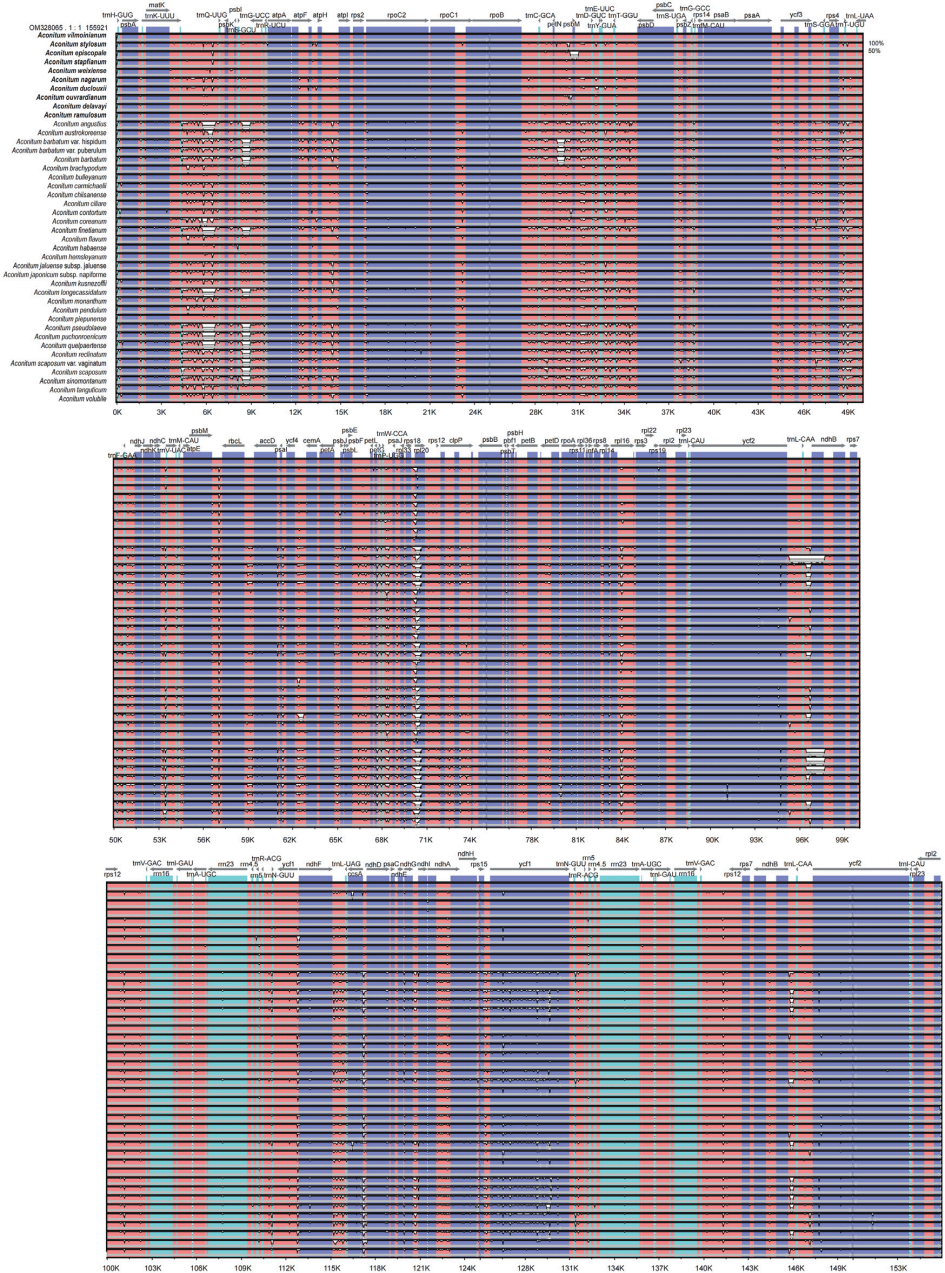
Global chloroplast genome alignments for 42 *Aconitum* species using the mVISTA program, with *A. vilmorinianum* as the reference. Y-axis shows the range of sequence identity (50–100%).

In this study, the chloroplast genome of *A*. *vilmorinianum* was used as the reference sequence to compare the chloroplast genome in pairs with that of the other 41 species. The Shuffle—LAGAN model of mVISTA was used to map the chloroplast genomes of 42 *Aconitum* species (Figure 9) and observe the approximate gene composition and sequence. The results showed that the chloroplast genome sequences of 42 *Aconitum* plants were similar, revealing that the chloroplast genome of *Aconitum* was highly conserved. The IR region of the *Aconitum* chloroplast genome was more conserved than LSC and SSC regions, the coding region was more conserved than the non-coding region, and the variation degree of the intergenic spacer region was greater than that of the gene region. The intergenic spacer region *trnK*-*UUU*-*trnQ*-*UUG*, *trnS-GCU-trnG-UCC*, *atpH-atpI*, *petN-psbM*, *rps18-rpl20*, *rpl16-rps3*, *trnL-CAA-trnL-CAA*, *ndhB-trnL-CAA* had the largest variation; The protein-coding gene region was the most conserved, and only *ycf1* and *ycf2* had the largest variation; Four rRNA genes (*rrn4.5*, *rrn5*, *rrn16,* and *rrn23*) were the most conserved. These highly variable genes and intergenic spacers can be used as molecular markers for 42 *Aconitum* species phylogenetic and population genetics studies.

### Nucleotide Polymorphism Analysis

**FIGURE 10:**
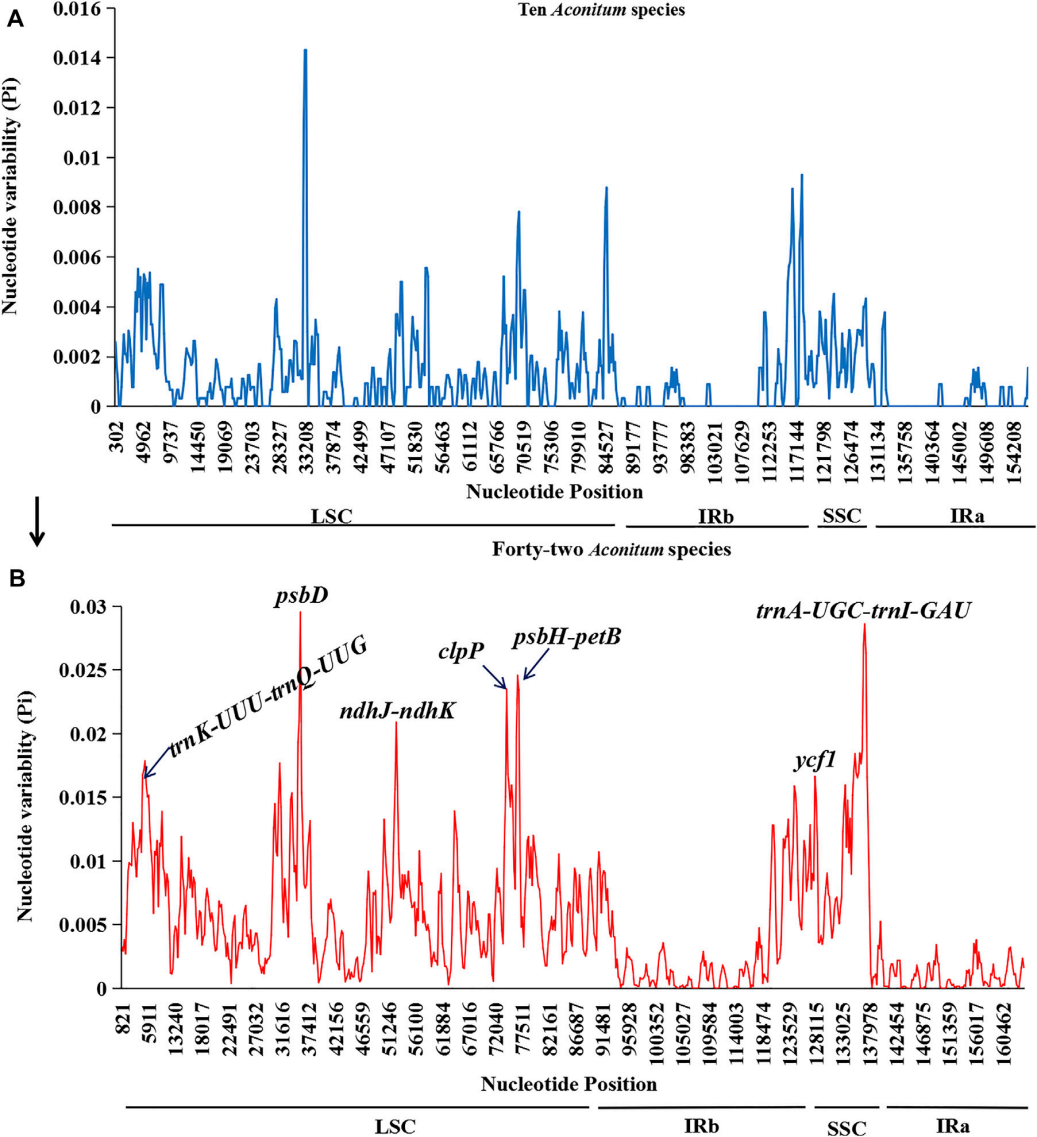
Sliding window analyses of the whole plastomes of *Aconitum* species. X-axis: position of the midpoint of a window, Y-axis: nucleotide diversity (Pi) of each window. **(A)**: Sliding window analysis of ten *Aconitum* species sequenced in this study. **(B)**: Sliding window analyses the whole plastomes of 42 *Aconitum* species (comprising ten species for this study).

The recognition of highly variable sites in the whole chloroplast genome can be used as molecular markers for species identification and phylogenetic studies (Li et al., 2019). This study screened the highly variable sites of 42 *Aconitum* species by sliding window analysis. Seven hotspots with high variation were screened from the chloroplast genome sequence, including four intergenic spacer regions and three protein-coding gene regions (Figure 10B). They were distributed in the LSC region (*trnK-UUU-trnQ-UGG*, *psbD*, *ndhJ-ndhK*, *clpP*, and *psbH-petB*) and SSC region (*ycf1* and *trnA-UGC-trnI-GAU*). The LSC region had the most variation sites, followed by the SSC region. In contrast, the IRb and IRa regions had relatively few variation sites. The distribution of Pi value was significantly lower than in LSC and SSC regions, which also revealed that in the chloroplast genome, the IRs region variation was conservative compared with LSC and SSC regions.

In addition, we performed chloroplast genome sliding window analyses of the ten *Aconitum* species included in this study (Figure 10A). We found that the variable sites were more divergent when variation analysis was performed on 42 *Aconitum* species. Analysis of the variant sites in all the 42 *Aconitum* species that have been sequenced allows screening of more suitable molecular identification markers for *Aconitum* species.

### Phylogenetic Analysis

**FIGURE 11:**
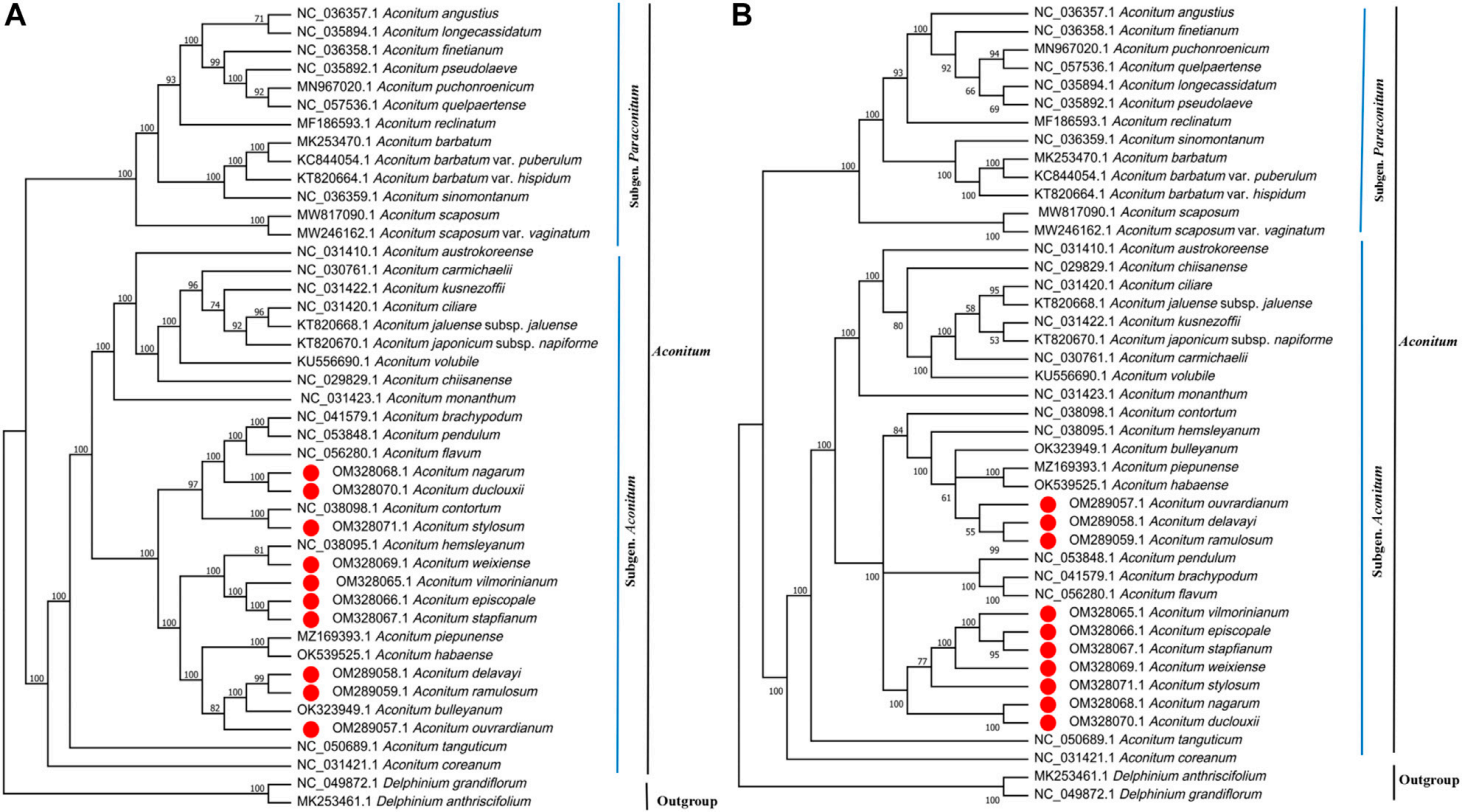
Phylogenetic relationship of 42 *Aconitum* species inferred from Maximum Likelihood (ML) based on two data sets. **(A)** ML tree constructed based on complete chloroplast genome. **(B)** ML tree constructed based on protein-coding genes (PCGs).

This study used Maximum Likelihood (ML) to construct phylogenetic trees based on two data sets (complete chloroplast genome and 84–85 protein-coding genes) from 44 species to determine the phylogenetic position of ten *Aconitum* species (Figures 11A,B). In addition, NJ tree with complete chloroplast genome sequences of 44 species were constructed. The topological structure of the NJ tree is essentially consistent with that of the ML tree (Supplementary Figure S1). The information on 44 species involved in phylogenetic tree construction is shown in Supplementary Table S6. The ML tree topology constructed based on the two data sets was essentially consistent, and a high support rate was detected on most nodes, but the support rate of the ML trees constructed based on different data sets was different (Figures 11A,B). The clustering of the two phylogenetic trees was the same, which strongly supports that *Aconitum* mainly consists of Subgen. *Aconitum* and Subgen. *ParAconitum*, with support values ≥100%. Two outgroup species (*Delphinium grandiflorum* and *Delphinium anthriscifolium*) were independent. Forty-two *Aconitum* species were clustered into a single large clade with support values ≥60%. Of these, *A. stapfianum*, *A. weixiense*, *A. vilmorinianum*, and *A. episcopale* were clustered together. *A. stapfianum* and *A. episcopal*e exhibited a sister relationship with 96% and 100% support values, indicating the close relationship between the four *Aconitum* species. *A. delavayi*, *A*. *ramulosum*, and *A. ouvrardianum* clustered together, with 98% and 100% support values. *A. delavayi* and *A*. *ramulosum* exhibited a sister relationship. However, *A*. *stylosum*, *A*. *stapfianum*, *A*. *weixiense*, *A*. *vilmorinianum*, and *A*. *episcopale* were clustered together in ML phylogenetic tree based on PCGs. Our results will provide genomic resources for the phylogeny of *Aconitum* species and facilitate future phylogenetic studies and other studies of Ranunculaceae plants.

## Discussion

The chloroplast genomes of *Nicotiana tabacum* (Shinozaki et al., 1986) and *Marchantia polymorpha* (Ohyama et al., 1986) were sequenced in 1986. As of November 2021, more than 6,500 chloroplast genomes have been recorded in the GeneBank database. The whole chloroplast genome is simple and easy to obtain (Badenes and Parfitt, 1995). Moreover, the chloroplast genome is more conservative and shorter than the nuclear and mitochondrial genome. It has often been used as a DNA super barcode for the identification, classification, and phylogenetic research of medicinal plants in recent years (Rønsted et al., 2005; Yuan, 2020). Hu et al. used the whole chloroplast genome as DNA super barcode to identify four medicinal plants of *Gentian* from Yunnan, and the results showed that DNA super barcode has excellent advantages in species identification of *Gentian* complex groups (Hu and Zhang, 2021). Cui et al. compared the whole genome sequences of 32 *Cardamom* species, revealing that the chloroplast whole genome can be used to accurately identify *Cardamom* species (Cui et al., 2019). Guo et al. determined the sister relationship between *Schisandra* and *Illicium* by constructing ML and BI phylogenetic trees (Guo et al., 2017). In this study, the chloroplast genomes of ten medicinal plants of *Aconitum* from Yunnan were sequenced in the next-generation sequencing. The genomic characteristics and composition of ten *Aconitum* species were analyzed. Moreover, we also performed a comparative analysis with other sequenced species in the GenBank database. Interspecific genetic studies and species identification of *Aconitum* species will be facilitated by chloroplast genome alignment, comparative analysis, and phylogenetic analysis.

*Aconitum* species are very diverse, and many species are highly similar in plant morphology and medicinal site roots, which are difficult to identify morphologically. Some authors have attempted to identify some *Aconitum* species with the DNA barcodes *ITS2* and *psbA*-*trnH* with some limitations and difficulty in making accurate identifications (Zhu et al., 2020). Park et al. (2017) found highly divergent regions, including *trnK*-*trnQ*, *ycf1*-*ndhF*, and *ycf4*-*cemA*, by comparing the chloroplast genomes of three *Aconitum* medicinal plants. Developed InDel markers based on the hypervariable region using indel sequences in *trnK*-*trnQ* and *ycf1*-*ndhF* and confirmed that *A. pseudolaeve*, *A. longecassidatum*, and *A. barbatum* could be clearly distinguished using novel InDel markers AcoTT (*Aconitum trnK*-*trnQ*) and AcoYN (*Aconitum ycf1-ndhF*). Therefore, molecular marker studies of chloroplast genomes of *Aconitum* species are relatively lacking. The ten *Aconitum* species used in this study have a long medicinal use history in Yunnan and surrounding areas. They have many applications in folk, such as Yi, Bai, Tibetan, and Naxi, and root tubers are often used to treat rheumatic arthritis pain, fall injury mainly, and so on (Jiang et al., 2016). However, several medicinal herbs are toxic, and mixed-use may endanger human life safety. Therefore, the addition of molecular studies to ten *Aconitum* species will benefit the identification and characterization of the species. Based on the ten *Aconitum* species sequenced in this study, genome comparison with other sequenced *Aconitum* species and sliding window analysis were performed to obtain seven highly divergent regions (*trnK-UUU-trnQ-UGG*, *ndhJ-ndhK*, *psbH-petB*, *trnA-UGC-trnI-GAU*, *psbD*, *clpP*, and *ycf1*). These highly divergent regions provide helpful information for molecular marker development in plant identification and investigating the phylogenetic relationships of *Aconitum*.

The chloroplast genome variation of *Aconitum* species was generally conserved. In the *Aconitum* chloroplast genome structure, the reverse repeat sequence (IRs) was separated by LSC and SSC (Mower and Vickrey, 2018). In different plants, the IRs region was highly conserved, the length ranging from 20,000 bp to 25,000 bp (Stewart A et al., 2019). In addition, intron sequences in chloroplast genomes were highly conserved. In this study, the IRs region was highly conservative compared to the LSC/SSC region, and the intron region was relatively conserved compared to the gene spacer region. The results of this study were consistent with the results of other scholars’ studies on the chloroplast genome of *Aconitum* species (Kong et al., 2017). It is also revealed that more highly variable segments could be mined from the chloroplast genome structure except the IRs and intron gene regions for inter-species identification.

Codon usage preference (CUB) is an important evolutionary feature in genomes and has been widely documented in many organisms, from prokaryotes to eukaryotes (Sharp et al., 1998). Some scholars analyzed the codons of chloroplast genomes of 19 *Panicum* species by bias and cluster analysis and revealed the overall evolutionary differences between chloroplast genomes of *Panicum* (Li et al., 2021). Wang et al. explored the codon usage preference of chloroplasts in the *Paris polyphylla* var. *yunnanensis*, providing a reference for heterologous expression and gene function in the *P. polyphylla* var. *yunnanensis* (Wang et al., 2021). Relative synonymous codon usage (RSCU) is an essential metric for determining codon usage preferences, and RSCU values are limited to 1; If RSCU >1, indicating a high frequency of codon usage; If RSCU <1, this codon is used less frequently; Or RSCU = 1, the codon usage is unbiased (Sharp et al., 1986). In the present study, the total GC content of the ten *Aconitum* chloroplast genomes was 38.61%. Of the 31 high-frequency codons with RSCU >1, 13 ended with A, 16 ended with U, one ended with G, and one ended with G. Our results showed that the third base of codon tended to end with A/U and the composition of bases at different positions of codons, which was consistent with the codon bias of other medicinal plants such as *Delphinium grandiflorum* (Duan et al., 2021). Some scholars have suggested that the codon usage pattern in plant genomes is closer to that of humans and higher eukaryotes than in other unicellular organisms, and this similarity is due to the overall preference of the third base of the codon for G+C content (Murray et al., 1988).

Pseudogenes play an essential role in gene expression regulation and genome evolution (Liu et al., 2010). Pseudogenes *ycf15*, *rps16*, *infA*, *rps19,* and *ycf1* were commonly found in the *Aconitum* genome (Kong et al., 2017; Meng et al., 2018). However, only two pseudogenes of *rps19* and *ycf1* were found in this study, and the *ycf1* was located in the IRb region. Some scholars have proved that their location caused pseudogenes *rps19* and *ycf1* at the boundary of each region of the chloroplast genome, and a repeat occurs at the boundary of IRs and SSC/LSC, leading to the incomplete gene in the IRs region, which was called boundary effect (Li et al., 2018). Pseudogene *rps19* was found only in *A. ouvrardianum*, *A. delavayi*, and *A*. *ramulosum* among the ten *Aconitum* species in this study, indicating specific differences in pseudogene phenomenon among species.

SSRs mainly distributed in the non-coding region, and the degree of sequence variation was higher than in the coding region (Powell et al., 1995). In addition, SSRs could be used to study conservation genetics of endangered plant species, molecular identification, and genetic relationships among related species (Clark et al., 2000; Huang et al., 2015). This study obtained 48–79 SSRs loci from 42 *Aconitum* species. Most of these SSRs were located in the LSC region, followed by the SSC and IR regions. The most abundant were mononucleotide repeats, which contributed to A/T richness. These results are consistent with most reported angiosperms (Nunzio et al., 2018; Gu et al., 2019; Tang et al., 2021). Analysis of the SSRs identified in the chloroplast genome of *Aconitum* showed some differences in the number of SSRs among the 42 species. Long repeats play an essential role in the complete chloroplast genome’s variation, expansion, and rearrangement (Asaf et al., 2017). We also identified 17–77 long repeats in the complete chloroplast genomes of 42 *Aconitum* species. The results suggest that long repeats were also divergent among different lineages. The SSRs and long repeats of the 42 *Aconitum* chloroplast genome exhibited abundant variation. They thus might help detect polymorphisms at the intraspecific level and develop molecular markers for *Aconitum* species for future evolutionary and genetic diversity studies.

We constructed the phylogenetic trees of 42 *Aconitum* species based on two data sets. There were no apparent conflicts between the phylogenetic trees constructed by different datasets, but the most support values of the branches based on the complete CP genomes dataset were higher than those based on the PCGs dataset. In addition, the sister relationships of *A. contortum* and *A. stylosum* changed in the ML phylogenetic tree constructed based on PCGs, and the *A. stylosum* was clustered with *A. stapfianum*, *A. weixiense*, *A. vilmorinianum*, and *A. episcopale* into one branch. The results suggest that determining the phylogenetic relationships of *Aconitum* species based on chloroplast whole genomes is more efficient than using PCGs alone. The ML phylogenetic trees based on the whole chloroplast genome and protein-coding gene sequences proved that *Aconitum* mainly consists of Subgen. *Aconitum* and Subgen. *ParAconitum,* the results were similar to phylogenetic studies on *Aconitum* by other scholars. (Meng et al., 2018; Kim et al., 2019). The genus *Aconitum* in *Flora of China* was classified into the Subgen. *Aconitum*, Subgen. *Gymnaconitum*, Subgen. *ParAconitum*, and the phylogenetic tree support subgenera classification in *Aconitum* spp. (Li and Kadota, 2001). In the phylogenetic tree constructed based on the whole chloroplast genome, the related species of *Aconitum* are clustered into different branches with Ser. *Ambigua*, Ser. *Brachypoda*, Ser. *Bullatifolia*, Ser. *Stylosa*, Ser. *Tangutica*, Ser. *Volubilia*. The results revealed that the related species of Ser. *Ambigua* (*A. piepunense*, *A. delavayi*, *A. ramulosum*, *A. ouvrardianum*) and Ser. *Volubilia* (*A. hemsleyanum*, *A. weixiense*, *A. vilmorinianum*, *A. episcopale*, *A. stapfianum*) are clustered into sister branches and have a close genetic relationship. Ser. *Brachypoda* (*A*. *brachypodum*, *A*. *pendulum*, *A. flavum*), Ser. *Bullatifolia* (*A. nagarum*, *A. duclouxii*), and Ser. *Stylosa* (*A*. *stylosum*, *A*. *contortum*) were clustered into a single clade with 97% support values, where Ser. *Bullatifolia* and Ser. *Stylosa* exhibited close phylogenetic relationships. It is worth noting that *A. habaense*, classified as the Ser. *Volubilia*, was clustered in the branch of the Ser. *Ambigua*. *A. tanguticum* was singly clustered as a branch of the Ser. *Tangutica*. Ten *Aconitum* species were sequenced in current were divided into four groups: Ser. *Volubilia*, Ser. *Stylosa*, Ser. *Ambigua*, and Ser. *Bullatifolia*. For the first time, a phylogenetic tree was constructed for *A*. *stylosum*, *A. nagarum*, *A. duclouxii*, *A. stapfianum*, *A. weixiense*, *A. ouvrardianum*, and *A. delavayi* with other *Aconitum* species. *A. delavayi* and *A. ramulosum* were sister specie; *A. episcopale* and *A. stapfianum* were sister species; *A. delavayi* and *A. ramulosum* as sister species. The complete chloroplast genome resolved the more complex phylogeny among *Aconitum* species, and it also shows a high support rate, indicating that the way to employ the chloroplast genome to explore the phylogenetic relationships among *Aconitum* species is effective.

## Conclusion

This study reported that the CP genomes from ten *Aconitum* species ranged from 155,475 (*A. stylosum*) to 155,921 bp (*A. vilmoinianum*), and the structure and composition of the CP genomes are highly similar. The CP genomes genes from 129 (*A. vilmoinianum*) to 132 (*A*. *ramulosum*), including 83–85 protein-coding genes, 37 tRNA genes, eight rRNA genes, and two pseudogenes. The RSCU values of 31 codons were greater than one, RSCU values of 31 codons were less than one, RSCU values of two codons were one, and the high-frequency codons preferred to end with the A/T base. 48–79 SSRs and 17–77 long repeats were identified in the chloroplast genome of 42 *Aconitum* species. The variation of IRs was more conservative than that of LSC and SSC. Four highly variable intergenic spacers (*trnK-UUU-trnQ-UGG*, *ndhJ-ndhK*, *psbH-petB*, and *trnA-UGC-trnI-GAU*) and three gene regions (*psbD*, *clpP*, and *ycf1*) can be used as appropriate DNA barcodes for species identification and genetic diversity studies of *Aconitum* species. Among the ten *Aconitum* species, *A. nagarum* had the closest relationship with *A*. *duclouxii*, *A*. *stapfianum* had the closest relationship with *A*. *episcopale*, *A. delavayi* had the closest relationship with *A*. *ramulosum*, respectively. This study enriched the complete chloroplast genome resources of *Aconitum* species and could provide scientific evidence for developing molecular markers, species identification, and phylogeny of *Aconitum* species.

## Data Availability

The datasets presented in this study can be found in online repositories. The names of the repository/repositories and accession number(s) can be found in the article/Supplementary Material.
