# Supplementary material for: Comparative Analysis of the Chloroplast Genome for Aconitum Species: Genome Structure and Phylogenetic Relationships
Source: Front Genet. 2022 May 31;13:878182. doi: 10.3389/fgene.2022.878182 (PMC9194378; doi:10.3389/fgene.2022.878182)
Supplement: Supplementary file 1 [file DataSheet1.docx]

Supplementary Material

**Table S1.** Base composition of chloroplast genomes of ten *Aconitum* species.

| **Species** | **Regions** | **Positions** | **T(U) (%)** | **C (%)** | **A (%)** | **G (%)** | **AT%** | **Length (bp)** |
| --- | --- | --- | --- | --- | --- | --- | --- | --- |
| 1. ***vilmorinianum*** | Total |  | 31.3 | 19.1 | 30.6 | 18.9 | 61.9 | 155,921 |
|  | IR |  | 28.5 | 21.5 | 28.5 | 21.5 | 57.0 | 52,472 |
|  | LSC |  | 32.5 | 18.5 | 31.4 | 17.7 | 63.8 | 86,524 |
|  | SSC |  | 34.1 | 15.2 | 33.3 | 17.4 | 67.4 | 16,925 |
|  | CDS |  | 31.1 | 17.8 | 30.5 | 20.5 | 61.7 | 79,014 |
|  | tRNA |  | 25.3 | 23.6 | 21.8 | 29.3 | 47.1 | 2,791 |
|  | rRNA |  | 18.5 | 23.7 | 25.9 | 31.8 | 44.5 | 8,652 |
|  |  | 1st position | 23.5 | 18.9 | 30.6 | 27.0 | 54.2 | 26,338 |
|  |  | 2nd position | 32.2 | 20.5 | 29.3 | 18.0 | 61.6 | 26,338 |
|  |  | 3rd position | 37.7 | 14.2 | 31.6 | 16.5 | 69.3 | 26,338 |
| 1. ***stylosum*** | Total |  | 31.3 | 19.2 | 30.6 | 19.0 | 61.9 | 155,475 |
|  | IR |  | 28.5 | 21.5 | 28.5 | 21.5 | 57.0 | 52,448 |
|  | LSC |  | 32.4 | 18.5 | 31.4 | 17.7 | 63.7 | 86,098 |
|  | SSC |  | 34.1 | 15.2 | 33.3 | 17.4 | 67.4 | 16,929 |
|  | CDS |  | 31.2 | 17.9 | 30.5 | 20.5 | 61.7 | 78,906 |
|  | tRNA |  | 25.3 | 23.6 | 21.8 | 29.3 | 47.0 | 2,791 |
|  | rRNA |  | 18.5 | 23.7 | 25.9 | 31.8 | 44.5 | 8,652 |
|  |  | 1st position | 23.5 | 18..9 | 30.6 | 27.0 | 54.1 | 26,302 |
|  |  | 2nd position | 32.3 | 20.5 | 29.3 | 18.0 | 61.6 | 26,302 |
|  |  | 3rd position | 37.7 | 14.2 | 31.6 | 16.5 | 69.3 | 26,302 |
| 1. ***episcopale*** | Total |  | 31.3 | 19.1 | 30.6 | 18.9 | 61.9 | 155,853 |
|  | IR |  | 28.5 | 21.5 | 28.5 | 21.5 | 57.0 | 86,443 |
|  | LSC |  | 32.5 | 18.5 | 31.4 | 17.7 | 63.8 | 16,938 |
|  | SSC |  | 34.1 | 15.2 | 33.3 | 17.4 | 67.4 | 52,472 |
|  | CDS |  | 31.1 | 17.8 | 30.5 | 20.5 | 61.7 | 2,791 |
|  | tRNA |  | 25.3 | 23.6 | 21.8 | 29.3 | 47.1 | 8,652 |
|  | rRNA |  | 18.5 | 23.7 | 25.9 | 31.8 | 44.5 | 78,996 |
|  |  | 1st position | 23.5 | 18.9 | 30.6 | 27.0 | 54.2 | 26,332 |
|  |  | 2nd position | 32.2 | 20.5 | 29.3 | 18.0 | 61.6 | 26,332 |
|  |  | 3rd position | 37.7 | 14.2 | 31.6 | 16.5 | 69.3 | 26,332 |
| 1. ***stapfianum*** | Total |  | 31.3 | 19.1 | 30.6 | 18.9 | 61.9 | 155,858 |
|  | IR |  | 28.5 | 21.5 | 28.5 | 21.5 | 57.0 | 86,449 |
|  | LSC |  | 32.5 | 18.5 | 31.4 | 17.7 | 63.8 | 16,937 |
|  | SSC |  | 34.1 | 15.2 | 33.3 | 17.4 | 67.4 | 52,472 |
|  | CDS |  | 31.1 | 17.8 | 30.5 | 20.5 | 61.7 | 2,791 |
|  | tRNA |  | 25.3 | 23.6 | 21.8 | 29.3 | 47.1 | 8,652 |
|  | rRNA |  | 18.5 | 23.7 | 25.9 | 31.8 | 44.5 | 78,996 |
|  |  | 1st position | 23.5 | 18.9 | 30.6 | 27.0 | 54.2 | 26,332 |
|  |  | 2nd position | 32.2 | 20.5 | 29.3 | 18.0 | 61.6 | 26,332 |
|  |  | 3rd position | 37.7 | 14.2 | 31.6 | 16.5 | 69.3 | 26,332 |
| 1. ***weixiense*** | Total |  | 31.3 | 19.1 | 30.6 | 18.9 | 61.9 | 155,872 |
|  | IR |  | 28.5 | 21.5 | 28.5 | 21.5 | 57.0 | 86,493 |
|  | LSC |  | 32.4 | 18.5 | 31.4 | 17.7 | 63.8 | 16,921 |
|  | SSC |  | 34.1 | 15.2 | 33.3 | 17.4 | 67.5 | 52,458 |
|  | CDS |  | 31.2 | 17.8 | 30.5 | 20.5 | 61.7 | 2,791 |
|  | tRNA |  | 25.3 | 23.6 | 21.8 | 29.3 | 47.1 | 8,652 |
|  | rRNA |  | 18.5 | 23.7 | 25.9 | 31.8 | 44.5 | 78,978 |
|  |  | 1st position | 23.5 | 18.9 | 30.6 | 27.0 | 54.2 | 26,326 |
|  |  | 2nd position | 32.2 | 20.5 | 29.3 | 18.0 | 61.6 | 26,326 |
|  |  | 3rd position | 37.7 | 14.2 | 31.6 | 16.5 | 69.3 | 26,326 |
| 1. ***nagarum*** | Total |  | 31.3 | 19.2 | 30.6 | 19.0 | 61.9 | 155,732 |
|  | IR |  | 28.5 | 21.5 | 28.5 | 21.5 | 57.0 | 86,313 |
|  | LSC |  | 32.4 | 18.5 | 31.4 | 17.7 | 63.8 | 16,983 |
|  | SSC |  | 34.1 | 15.2 | 33.3 | 17.4 | 67.4 | 52,436 |
|  | CDS |  | 31.2 | 17.9 | 30.5 | 20.5 | 61.7 | 2,791 |
|  | tRNA |  | 25.3 | 23.6 | 21.8 | 29.3 | 47.1 | 8,652 |
|  | rRNA |  | 18.5 | 23.7 | 25.9 | 31.8 | 44.5 | 78,984 |
|  |  | 1st position | 23.5 | 18.9 | 30.6 | 27.0 | 54.1 | 26,328 |
|  |  | 2nd position | 32.2 | 20.5 | 29.3 | 17.9 | 61.6 | 26,328 |
|  |  | 3rd position | 37.7 | 14.2 | 31.6 | 16.5 | 69.3 | 26,328 |
| 1. ***duclouxii*** | Total |  | 31.3 | 19.2 | 30.6 | 19.0 | 61.9 | 155,479 |
|  | IR |  | 28.5 | 21.5 | 28.5 | 21.5 | 56.9 | 86,318 |
|  | LSC |  | 32.4 | 18.5 | 31.4 | 17.7 | 63.8 | 16,943 |
|  | SSC |  | 34.0 | 15.3 | 33.3 | 17.5 | 67.3 | 52,218 |
|  | CDS |  | 31.2 | 17.9 | 30.5 | 20.5 | 61.7 | 2,791 |
|  | tRNA |  | 25.3 | 23.6 | 21.8 | 29.3 | 47.0 | 8,652 |
|  | rRNA |  | 18.5 | 23.7 | 25.9 | 31.8 | 44.5 | 78,891 |
|  |  | 1st position | 23.5 | 18.9 | 30.6 | 27.0 | 54.1 | 26,297 |
|  |  | 2nd position | 32.3 | 20.5 | 29.3 | 17.9 | 61.6 | 26,297 |
|  |  | 3rd position | 37.7 | 14.2 | 31.6 | 16.5 | 69.3 | 26,297 |
| 1. ***ouvrardianum*** | Total |  | 31.2 | 19.4 | 30.7 | 18.7 | 61.9 | 155,799 |
|  | IR |  | 28.5 | 21.5 | 28.5 | 21.5 | 57.0 | 86,420 |
|  | LSC |  | 32.4 | 18.5 | 31.4 | 17.7 | 63.8 | 16,903 |
|  | SSC |  | 33.3 | 17.4 | 34.1 | 15.2 | 67.4 | 52,476 |
|  | CDS |  | 31.1 | 17.9 | 30.5 | 20.5 | 61.6 | 2,812 |
|  | tRNA |  | 25.2 | 23.5 | 21.9 | 29.3 | 47.1 | 9,050 |
|  | rRNA |  | 18.6 | 23.8 | 25.8 | 31.8 | 44.5 | 78,324 |
|  |  | 1st position | 23.5 | 18.9 | 30.6 | 27.0 | 54.1 | 26,108 |
|  |  | 2nd position | 32.2 | 20.5 | 29.3 | 18.0 | 61.5 | 26,108 |
|  |  | 3rd position | 37.7 | 14.2 | 31.6 | 16.5 | 69.3 | 26,108 |
| 1. ***delavayi*** | Total |  | 31.2 | 19.4 | 30.7 | 18.7 | 61.9 | 155,733 |
|  | IR |  | 28.5 | 21.5 | 28.5 | 21.5 | 57.0 | 86,362 |
|  | LSC |  | 32.4 | 18.5 | 31.4 | 17.7 | 63.8 | 16,913 |
|  | SSC |  | 33.3 | 17.4 | 34.1 | 15.2 | 67.4 | 52,458 |
|  | CDS |  | 31.1 | 17.9 | 30.5 | 20.5 | 61.6 | 2,812 |
|  | tRNA |  | 25.2 | 23.5 | 21.9 | 29.3 | 47.1 | 9,050 |
|  | rRNA |  | 18.6 | 23.8 | 25.8 | 31.8 | 44.5 | 78,258 |
|  |  | 1st position | 23.5 | 18.9 | 30.6 | 27.0 | 54.1 | 26,086 |
|  |  | 2nd position | 32.2 | 20.5 | 29.3 | 18.0 | 61.5 | 26,086 |
|  |  | 3rd position | 37.7 | 14.2 | 31.6 | 16.5 | 69.3 | 26,086 |
| 1. ***ramulosum*** | Total |  | 31.2 | 19.4 | 30.7 | 18.7 | 61.9 | 155,841 |
|  | IR |  | 28.5 | 21.5 | 28.5 | 21.5 | 57.0 | 86,470 |
|  | LSC |  | 32.4 | 18.5 | 31.4 | 17.7 | 63.8 | 16,913 |
|  | SSC |  | 33.3 | 17.4 | 34.1 | 15.2 | 67.4 | 52,458 |
|  | CDS |  | 31.1 | 17.9 | 30.5 | 20.5 | 61.6 | 2,812 |
|  | tRNA |  | 25.2 | 23.5 | 21.9 | 29.3 | 47.1 | 9,050 |
|  | rRNA |  | 18.6 | 23.8 | 25.8 | 31.8 | 44.5 | 78,366 |
|  |  | 1st position | 23.5 | 18.9 | 30.6 | 27.0 | 54.1 | 26,122 |
|  |  | 2nd position | 32.2 | 20.5 | 29.3 | 18.0 | 61.5 | 26,122 |
|  |  | 3rd position | 37.7 | 14.2 | 31.6 | 16.5 | 69.3 | 26,122 |

**Table S2.** Location and length of chloroplast genes containing introns in *Aconitum.*

| ***Aconitum vilmorinianum*** | | | | | | |
| --- | --- | --- | --- | --- | --- | --- |
| Gene | Location | Length (bp) | | | | |
|  |  | Exon1 (bp) | Intron1 (bp) | Exon2 (bp) | Intron2 (bp) | Exon3 (bp) |
| *atpF* | LSC | 410 | 735 | 145 |  |  |
| *clpP* | LSC | 246 | 674 | 292 | 847 | 71 |
| *ndhA* | SSC | 553 | 1,012 | 539 |  |  |
| *ndhB* | IRB | 756 | 706 | 777 |  |  |
| *ndhB* | IRA | 777 | 706 | 756 |  |  |
| *petB* | LSC | 6 | 798 | 642 |  |  |
| *petD* | LSC | 8 | 715 | 475 |  |  |
| *rpl16* | LSC | 399 | 1,107 | 9 |  |  |
| *rpl2* | IRB | 434 | 665 | 391 |  |  |
| *rpl2* | IRA | 391 | 665 | 434 |  |  |
| *rpoC1* | LSC | 1,611 | 746 | 432 |  |  |
| *rps12* | - | 114 | 69,975 | 232 | 542 | 26 |
| *rps12* | - | 114 | 28,132 | 26 | 542 | 232 |
| *trnA-UGC* | IRB | 38 | 800 | 35 |  |  |
| *trnA-UGC* | IRA | 35 | 800 | 38 |  |  |
| *trnG-UCC* | LSC | 23 | 719 | 48 |  |  |
| *trnI-GAU* | IRB | 37 | 940 | 35 |  |  |
| *trnI-GAU* | IRA | 35 | 940 | 37 |  |  |
| *trnK-UUU* | LSC | 35 | 2,520 | 37 |  |  |
| *trnL-UAA* | LSC | 35 | 493 | 50 |  |  |
| *trnV-UAC* | LSC | 35 | 592 | 39 |  |  |
| *ycf3* | LSC | 153 | 755 | 230 | 720 | 124 |
| ***Aconitum stylosum*** | | | | | | |
| Gene | Location | Length (bp) | | | | |
|  |  | Exon1 (bp) | Intron1 (bp) | Exon2 (bp) | Intron2 (bp) | Exon3 (bp) |
| *atpF* | LSC | 410 | 735 | 145 |  |  |
| *clpP* | LSC | 246 | 670 | 292 | 840 | 71 |
| *ndhA* | SSC | 553 | 1,006 | 539 |  |  |
| *ndhB* | IRB | 756 | 706 | 777 |  |  |
| *ndhB* | IRA | 777 | 706 | 756 |  |  |
| *petB* | LSC | 6 | 798 | 642 |  |  |
| *petD* | LSC | 8 | 715 | 475 |  |  |
| *rpl16* | LSC | 399 | 1,110 | 9 |  |  |
| *rpl2* | IRB | 434 | 665 | 391 |  |  |
| *rpl2* | IRA | 391 | 665 | 434 |  |  |
| *rpoC1* | LSC | 1,611 | 765 | 432 |  |  |
| *rps12* | - | 114 | 69,949 | 232 | 542 | 26 |
| *rps12* | - | 114 | 28,126 | 26 | 542 | 232 |
| *trnA-UGC* | IRB | 38 | 800 | 35 |  |  |
| *trnA-UGC* | IRA | 35 | 800 | 38 |  |  |
| *trnG-UCC* | LSC | 23 | 721 | 48 |  |  |
| *trnI-GAU* | IRB | 37 | 940 | 35 |  |  |
| *trnI-GAU* | IRA | 35 | 940 | 37 |  |  |
| *trnK-UUU* | LSC | 35 | 2,529 | 37 |  |  |
| *trnL-UAA* | LSC | 35 | 493 | 50 |  |  |
| *trnV-UAC* | LSC | 35 | 591 | 39 |  |  |
| *ycf3* | LSC | 153 | 756 | 230 | 720 | 124 |
| ***Aconitum episcopale*** | | | | | | |
| Gene | Location | Length (bp) | | | | |
|  |  | Exon1 (bp) | Intron1 (bp) | Exon2 (bp) | Intron2 (bp) | Exon3 (bp) |
| *atpF* | LSC | 410 | 734 | 145 |  |  |
| *clpP* | LSC | 246 | 673 | 292 | 847 | 71 |
| *ndhA* | SSC | 553 | 1,012 | 539 |  |  |
| *ndhB* | IRB | 756 | 706 | 777 |  |  |
| *ndhB* | IRA | 777 | 706 | 756 |  |  |
| *petB* | LSC | 6 | 798 | 642 |  |  |
| *petD* | LSC | 8 | 715 | 475 |  |  |
| *rpl16* | LSC | 399 | 1,107 | 9 |  |  |
| *rpl2* | IRB | 434 | 665 | 391 |  |  |
| *rpl2* | IRA | 391 | 665 | 434 |  |  |
| *rpoC1* | LSC | 1,611 | 746 | 432 |  |  |
| *rps12* | - | 114 | 69,997 | 232 | 542 | 26 |
| *rps12* | - | 114 | 28,141 | 26 | 542 | 232 |
| *trnA-UGC* | IRB | 38 | 800 | 35 |  |  |
| *trnA-UGC* | IRA | 35 | 800 | 38 |  |  |
| *trnG-UCC* | LSC | 23 | 719 | 48 |  |  |
| *trnI-GAU* | IRB | 37 | 940 | 35 |  |  |
| *trnI-GAU* | IRA | 35 | 940 | 37 |  |  |
| *trnK-UUU* | LSC | 35 | 2,521 | 37 |  |  |
| *trnL-UAA* | LSC | 35 | 493 | 50 |  |  |
| *trnV-UAC* | LSC | 35 | 592 | 39 |  |  |
| *ycf3* | LSC | 153 | 755 | 230 | 720 | 124 |
| ***Aconitum stapfianum*** | | | | | | |
| Gene | Location | Length (bp) | | | | |
|  |  | Exon1 (bp) | Intron1 (bp) | Exon2 (bp) | Intron2 (bp) | Exon3 (bp) |
| *atpF* | LSC | 410 | 734 | 145 |  |  |
| *clpP* | LSC | 246 | 673 | 292 | 847 | 71 |
| *ndhA* | SSC | 553 | 1,012 | 539 |  |  |
| *ndhB* | IRB | 756 | 706 | 777 |  |  |
| *ndhB* | IRA | 777 | 706 | 756 |  |  |
| *petB* | LSC | 6 | 798 | 642 |  |  |
| *petD* | LSC | 8 | 715 | 475 |  |  |
| *rpl16* | LSC | 399 | 1,107 | 9 |  |  |
| *rpl2* | IRB | 434 | 665 | 391 |  |  |
| *rpl2* | IRA | 391 | 665 | 434 |  |  |
| *rpoC1* | LSC | 1,611 | 746 | 432 |  |  |
| *rps12* | - | 114 | 69,986 | 232 | 542 | 26 |
| *rps12* | - | 114 | 28,131 | 26 | 542 | 232 |
| *trnA-UGC* | IRB | 38 | 800 | 35 |  |  |
| *trnA-UGC* | IRA | 35 | 800 | 38 |  |  |
| *trnG-UCC* | LSC | 23 | 719 | 48 |  |  |
| *trnI-GAU* | IRB | 37 | 940 | 35 |  |  |
| *trnI-GAU* | IRA | 35 | 940 | 37 |  |  |
| *trnK-UUU* | LSC | 35 | 2,521 | 37 |  |  |
| *trnL-UAA* | LSC | 35 | 493 | 50 |  |  |
| *trnV-UAC* | LSC | 35 | 592 | 39 |  |  |
| *ycf3* | LSC | 153 | 755 | 230 | 720 | 124 |
| ***Aconitum weixiense*** | | | | | | |
| Gene | Location | Length (bp) | | | | |
|  |  | Exon1 (bp) | Intron1 (bp) | Exon2 (bp) | Intron2 (bp) | Exon3 (bp) |
| *atpF* | LSC | 410 | 734 | 145 |  |  |
| *clpP* | LSC | 246 | 674 | 292 | 847 | 71 |
| *ndhA* | SSC | 553 | 1,007 | 539 |  |  |
| *ndhB* | IRB | 756 | 706 | 777 |  |  |
| *ndhB* | IRA | 777 | 706 | 756 |  |  |
| *petB* | LSC | 6 | 797 | 642 |  |  |
| *petD* | LSC | 8 | 715 | 475 |  |  |
| *rpl16* | LSC | 399 | 1,107 | 9 |  |  |
| *rpl2* | IRB | 434 | 665 | 391 |  |  |
| *rpl2* | IRA | 391 | 665 | 434 |  |  |
| *rpoC1* | LSC | 1,611 | 746 | 432 |  |  |
| *rps12* | - | 114 | 69,954 | 232 | 542 | 26 |
| *rps12* | - | 114 | 28,129 | 26 | 542 | 232 |
| *trnA-UGC* | IRB | 38 | 800 | 35 |  |  |
| *trnA-UGC* | IRA | 35 | 800 | 38 |  |  |
| *trnG-UCC* | LSC | 23 | 719 | 48 |  |  |
| *trnI-GAU* | IRB | 37 | 941 | 35 |  |  |
| *trnI-GAU* | IRA | 35 | 941 | 37 |  |  |
| *trnK-UUU* | LSC | 35 | 2,522 | 37 |  |  |
| *trnL-UAA* | LSC | 35 | 493 | 50 |  |  |
| *trnV-UAC* | LSC | 35 | 593 | 39 |  |  |
| *ycf3* | LSC | 153 | 755 | 230 | 720 | 124 |
| ***Aconitum nagarum*** | | | | | | |
| Gene | Location | Length (bp) | | | | |
|  |  | Exon1 (bp) | Intron1 (bp) | Exon2 (bp) | Intron2 (bp) | Exon3 (bp) |
| *atpF* | LSC | 410 | 771 | 145 |  |  |
| *clpP* | LSC | 246 | 669 | 292 | 840 | 71 |
| *ndhA* | SSC | 553 | 1,010 | 539 |  |  |
| *ndhB* | IRB | 756 | 706 | 777 |  |  |
| *ndhB* | IRA | 777 | 706 | 756 |  |  |
| *petB* | LSC | 6 | 798 | 642 |  |  |
| *petD* | LSC | 8 | 715 | 475 |  |  |
| *rpl16* | LSC | 399 | 1,117 | 9 |  |  |
| *rpl2* | IRB | 434 | 665 | 391 |  |  |
| *rpl2* | IRA | 391 | 665 | 434 |  |  |
| *rpoC1* | LSC | 1,617 | 745 | 432 |  |  |
| *rps12* | - | 114 | 69,989 | 232 | 542 | 26 |
| *rps12* | - | 114 | 28,124 | 26 | 542 | 232 |
| *trnA-UGC* | IRB | 38 | 800 | 35 |  |  |
| *trnA-UGC* | IRA | 35 | 800 | 38 |  |  |
| *trnG-UCC* | LSC | 23 | 721 | 48 |  |  |
| *trnI-GAU* | IRB | 37 | 940 | 35 |  |  |
| *trnI-GAU* | IRA | 35 | 940 | 37 |  |  |
| *trnK-UUU* | LSC | 35 | 2,520 | 37 |  |  |
| *trnL-UAA* | LSC | 35 | 493 | 50 |  |  |
| *trnV-UAC* | LSC | 35 | 592 | 39 |  |  |
| *ycf3* | LSC | 153 | 755 | 230 | 720 | 124 |
| ***Aconitum duclouxii*** | | | | | | |
| Gene | Location | Length (bp) | | | | |
|  |  | Exon1 (bp) | Intron1 (bp) | Exon2 (bp) | Intron2 (bp) | Exon3 (bp) |
| *atpF* | LSC | 410 | 771 | 145 |  |  |
| *clpP* | LSC | 246 | 670 | 292 | 840 | 71 |
| *ndhA* | SSC | 553 | 1,010 | 539 |  |  |
| *ndhB* | IRB | 756 | 706 | 777 |  |  |
| *ndhB* | IRA | 777 | 706 | 756 |  |  |
| *petB* | LSC | 6 | 798 | 642 |  |  |
| *petD* | LSC | 8 | 715 | 475 |  |  |
| *rpl16* | LSC | 399 | 1,116 | 9 |  |  |
| *rpl2* | IRB | 434 | 665 | 391 |  |  |
| *rpl2* | IRA | 391 | 665 | 434 |  |  |
| *rpoC1* | LSC | 1,617 | 745 | 432 |  |  |
| *rps12* | - | 114 | 69,732 | 232 | 542 | 26 |
| *rps12* | - | 114 | 28,125 | 26 | 542 | 232 |
| *trnA-UGC* | IRB | 38 | 800 | 35 |  |  |
| *trnA-UGC* | IRA | 35 | 800 | 38 |  |  |
| *trnG-UCC* | LSC | 23 | 722 | 48 |  |  |
| *trnI-GAU* | IRB | 37 | 940 | 35 |  |  |
| *trnI-GAU* | IRA | 35 | 940 | 37 |  |  |
| *trnK-UUU* | LSC | 35 | 2,520 | 37 |  |  |
| *trnL-UAA* | LSC | 35 | 493 | 50 |  |  |
| *trnV-UAC* | LSC | 35 | 592 | 39 |  |  |
| *ycf3* | LSC | 153 | 755 | 230 | 720 | 124 |
| ***Aconitum ouvrardianum*** | | | | | | |
| Gene | Location | Length (bp) | | | | |
|  |  | Exon1 (bp) | Intron1 (bp) | Exon2 (bp) | Intron2 (bp) | Exon3 (bp) |
| *atpF* | LSC | 410 | 734 | 145 |  |  |
| *clpP* | LSC | 246 | 672 | 291 | 850 | 69 |
| *ndhA* | SSC | 540 | 1,007 | 552 |  |  |
| *ndhB* | IRB | 756 | 706 | 777 |  |  |
| *ndhB* | IRA | 777 | 706 | 756 |  |  |
| *petB* | LSC | 6 | 798 | 642 |  |  |
| *petD* | LSC | 8 | 714 | 496 |  |  |
| *rpl16* | LSC | 399 | 1,093 | 9 |  |  |
| *rpl2* | IRB | 435 | 662 | 393 |  |  |
| *rpl2* | IRA | 393 | 662 | 435 |  |  |
| *rpoC1* | LSC | 1,620 | 734 | 435 |  |  |
| *rps12* | - | 114 | 69,934 | 232 | 542 | 26 |
| *rps12* | - | 26 | 28,117 | 232 | 542 | 114 |
| *trnA-UGC* | IRB | 38 | 805 | 35 |  |  |
| *trnA-UGC* | IRA | 35 | 805 | 38 |  |  |
| *trnG-GCC* | LSC | 23 | 720 | 47 |  |  |
| *trnI-GAU* | IRB | 42 | 935 | 35 |  |  |
| *trnI-GAU* | IRA | 35 | 935 | 42 |  |  |
| *trnK-UUU* | LSC | 35 | 2,521 | 37 |  |  |
| *trnL-UAA* | LSC | 35 | 493 | 50 |  |  |
| *trnV-UAC* | LSC | 37 | 590 | 39 |  |  |
| *ycf3* | LSC | 153 | 755 | 228 | 720 | 126 |
| ***Aconitum delavayi*** | | | | | | |
| Gene | Location | Length (bp) | | | | |
|  |  | Exon1 (bp) | Intron1 (bp) | Exon2 (bp) | Intron2 (bp) | Exon3 (bp) |
| *atpF* | LSC | 410 | 735 | 145 |  |  |
| *clpP* | LSC | 246 | 673 | 291 | 850 | 69 |
| *ndhA* | SSC | 540 | 1,007 | 552 |  |  |
| *ndhB* | IRB | 756 | 706 | 777 |  |  |
| *ndhB* | IRA | 777 | 706 | 756 |  |  |
| *petB* | LSC | 6 | 798 | 642 |  |  |
| *petD* | LSC | 8 | 714 | 496 |  |  |
| *rpl16* | LSC | 399 | 1,108 | 9 |  |  |
| *rpl2* | IRB | 435 | 662 | 393 |  |  |
| *rpl2* | IRA | 393 | 662 | 435 |  |  |
| *rpoC1* | LSC | 1,620 | 734 | 435 |  |  |
| *rps12* | - | 114 | 69,948 | 232 | 542 | 26 |
| *rps12* | - | 26 | 28,131 | 232 | 542 | 114 |
| *trnA-UGC* | IRB | 38 | 800 | 35 |  |  |
| *trnA-UGC* | IRA | 35 | 800 | 38 |  |  |
| *trnG-GCC* | LSC | 23 | 721 | 47 |  |  |
| *trnI-GAU* | IRB | 42 | 935 | 35 |  |  |
| *trnI-GAU* | IRA | 35 | 935 | 42 |  |  |
| *trnK-UUU* | LSC | 35 | 2,520 | 37 |  |  |
| *trnL-UAA* | LSC | 35 | 493 | 50 |  |  |
| *trnV-UAC* | LSC | 37 | 590 | 39 |  |  |
| *ycf3* | LSC | 153 | 755 | 228 | 720 | 126 |
| ***Aconitum ramulosum*** | | | | | | |
| Gene | Location | Length (bp) | | | | |
|  |  | Exon1 (bp) | Intron1 (bp) | Exon2 (bp) | Intron2 (bp) | Exon3 (bp) |
| *atpF* | LSC | 410 | 735 | 145 |  |  |
| *clpP* | LSC | 246 | 673 | 291 | 850 | 69 |
| *ndhA* | SSC | 540 | 1,007 | 552 |  |  |
| *ndhB* | IRB | 756 | 706 | 777 |  |  |
| *ndhB* | IRA | 777 | 706 | 756 |  |  |
| *petB* | LSC | 6 | 798 | 642 |  |  |
| *petD* | LSC | 8 | 714 | 496 |  |  |
| *rpl16* | LSC | 399 | 1,108 | 9 |  |  |
| *rpl2* | IRB | 435 | 662 | 393 |  |  |
| *rpl2* | IRA | 393 | 662 | 435 |  |  |
| *rpoC1* | LSC | 1,620 | 734 | 435 |  |  |
| *rps12* | - | 114 | 69,948 | 232 | 542 | 26 |
| *rps12* | - | 26 | 28,131 | 232 | 542 | 114 |
| *trnA-UGC* | IRB | 38 | 800 | 35 |  |  |
| *trnA-UGC* | IRA | 35 | 800 | 38 |  |  |
| *trnG-GCC* | LSC | 23 | 721 | 47 |  |  |
| *trnI-GAU* | IRB | 42 | 935 | 35 |  |  |
| *trnI-GAU* | IRA | 35 | 935 | 42 |  |  |
| *trnK-UUU* | LSC | 35 | 2,520 | 37 |  |  |
| *trnL-UAA* | LSC | 35 | 493 | 50 |  |  |
| *trnV-UAC* | LSC | 37 | 590 | 39 |  |  |
| *ycf3* | LSC | 153 | 755 | 228 | 720 | 126 |

**Table S3.** Frequency of amino acids in coding regions.

| **Species** | **Ala** | **Cys** | **Asp** | **Glu** | **Phe** | **Gly** | **His** | **Ile** | **Lys** | **Leu** | **Met** | **Asn** | **Pro** | **Gln** | **Arg** | **Ser** | **Thr** | **Val** | **Trp** | **Tyr** | **Total** |
| --- | --- | --- | --- | --- | --- | --- | --- | --- | --- | --- | --- | --- | --- | --- | --- | --- | --- | --- | --- | --- | --- |
| *Aconitum vilmorinianum* | 5.33 | 1.19 | 4.21 | 5.19 | 5.56 | 6.90 | 2.57 | 8.49 | 5.25 | 10.38 | 2.40 | 4.89 | 4.30 | 3.45 | 6.13 | 7.66 | 5.20 | 5.47 | 1.79 | 3.66 | 26,298 |
| *Aconitum stylosum* | 5.34 | 1.18 | 4.20 | 5.19 | 5.55 | 6.88 | 2.56 | 8.49 | 5.24 | 10.41 | 2.40 | 4.85 | 4.30 | 3.47 | 6.15 | 7.66 | 5.18 | 5.49 | 1.79 | 3.67 | 26,262 |
| *Aconitum episcopale* | 5.33 | 1.18 | 4.21 | 5.19 | 5.56 | 6.90 | 2.57 | 8.49 | 5.25 | 10.38 | 2.40 | 4.88 | 4.30 | 3.45 | 6.13 | 7.66 | 5.20 | 5.47 | 1.79 | 3.66 | 26,292 |
| *Aconitum stapfianum* | 5.33 | 1.18 | 4.21 | 5.19 | 5.56 | 6.90 | 2.57 | 8.49 | 5.25 | 10.38 | 2.40 | 4.88 | 4.30 | 3.45 | 6.13 | 7.66 | 5.20 | 5.47 | 1.79 | 3.66 | 26,292 |
| *Aconitum weixiense* | 5.33 | 1.18 | 4.21 | 5.19 | 5.56 | 6.89 | 2.56 | 8.49 | 5.24 | 10.39 | 2.40 | 4.88 | 4.30 | 3.45 | 6.15 | 7.66 | 5.19 | 5.47 | 1.79 | 3.67 | 26,286 |
| *Aconitum nagarum* | 5.33 | 1.18 | 4.20 | 5.20 | 5.58 | 6.87 | 2.56 | 8.48 | 5.23 | 10.40 | 2.40 | 4.87 | 4.30 | 3.47 | 6.14 | 7.67 | 5.19 | 5.48 | 1.79 | 3.67 | 26,288 |
| *Aconitum duclouxii* | 5.34 | 1.18 | 4.20 | 5.20 | 5.58 | 6.88 | 2.56 | 8.48 | 5.23 | 10.41 | 2.39 | 4.87 | 4.30 | 3.46 | 6.14 | 7.66 | 5.18 | 5.49 | 1.79 | 3.66 | 26,257 |
| *Aconitum ouvrardianum* | 5.38 | 1.18 | 4.20 | 5.16 | 5.51 | 6.92 | 2.58 | 8.52 | 5.22 | 10.37 | 2.40 | 4.89 | 4.33 | 3.46 | 6.12 | 7.63 | 5.18 | 5.47 | 1.82 | 3.64 | 26,067 |
| *Aconitum delavayi* | 5.38 | 1.18 | 4.20 | 5.16 | 5.51 | 6.90 | 2.58 | 8.52 | 5.21 | 10.39 | 2.40 | 4.88 | 4.34 | 3.46 | 6.12 | 7.64 | 5.18 | 5.48 | 1.82 | 3.64 | 26,045 |
| *Aconitum ramulosum* | 5.37 | 1.18 | 4.21 | 5.16 | 5.51 | 6.93 | 2.58 | 8.51 | 5.23 | 10.37 | 2.40 | 4.90 | 4.33 | 3.46 | 6.12 | 7.63 | 5.19 | 5.47 | 1.82 | 3.64 | 26,081 |

**Table S4.** RSCU analysis of each amino acid codon in the chloroplast genome of *Aconitum* species.

| **species** | **Amino acid** | **Codon** | **Number** | **RSCU value** | **Amino acid** | **Codon** | **Number** | **RSCU value** |
| --- | --- | --- | --- | --- | --- | --- | --- | --- |
| *Aconitum vilmorinianum* | Phe | UUU | 932 | 1.28 | Ser | UCU | 562 | 1.67 |
|  |  | UUC | 527 | 0.72 |  | UCC | 341 | 1.02 |
|  | Leu | UUA | 826 | 1.82 |  | UCA | 413 | 1.23 |
|  |  | UUG | 576 | 1.27 |  | UCG | 192 | 0.57 |
|  |  | CUU | 564 | 1.24 | Pro | CCU | 429 | 1.52 |
|  |  | CUC | 193 | 0.42 |  | CCC | 219 | 0.78 |
|  |  | CUA | 378 | 0.83 |  | CCA | 333 | 1.18 |
|  |  | CUG | 189 | 0.42 |  | CCG | 149 | 0.53 |
|  | Ile | AUU | 1074 | 1.45 | Thr | ACU | 530 | 1.55 |
|  |  | AUC | 457 | 0.62 |  | ACC | 257 | 0.75 |
|  |  | AUA | 697 | 0.94 |  | ACA | 429 | 1.26 |
|  | Met | AUG | 630 | 1 |  | ACG | 151 | 0.44 |
|  | Val | GUU | 527 | 1.47 | Ala | GCU | 594 | 1.71 |
|  |  | GUC | 162 | 0.45 |  | GCC | 228 | 0.66 |
|  |  | GUA | 547 | 1.52 |  | GCA | 393 | 1.13 |
|  |  | GUG | 202 | 0.56 |  | GCG | 177 | 0.51 |
|  | Tyr | UAU | 765 | 1.59 | Cys | UGU | 225 | 1.47 |
|  |  | UAC | 198 | 0.41 |  | UGC | 82 | 0.53 |
|  | TER | UAA | 38 | 1.37 | Trp | UGG | 470 | 1 |
|  |  | UAG | 25 | 0.9 | Arg | CGU | 358 | 1.33 |
|  |  | UGA | 20 | 0.72 |  | CGC | 93 | 0.35 |
|  | His | CAU | 509 | 1.51 |  | CGA | 363 | 1.35 |
|  |  | CAC | 164 | 0.49 |  | CGG | 118 | 0.44 |
|  | Gln | CAA | 684 | 1.51 |  | AGA | 499 | 1.86 |
|  |  | CAG | 222 | 0.49 |  | AGG | 182 | 0.68 |
|  | Asn | AAU | 992 | 1.55 | Ser | AGU | 392 | 1.17 |
|  |  | AAC | 290 | 0.45 |  | AGC | 114 | 0.34 |
|  | Lys | AAA | 990 | 1.44 | Gly | GGU | 601 | 1.33 |
|  |  | AAG | 388 | 0.56 |  | GGC | 185 | 0.41 |
|  | Asp | GAU | 882 | 1.6 |  | GGA | 723 | 1.6 |
|  |  | GAC | 222 | 0.4 |  | GGG | 304 | 0.67 |
|  | Glu | GAA | 996 | 1.46 |  |  |  |  |
|  |  | GAG | 366 | 0.54 |  |  |  |  |
| *Aconitum stylosum* | Phe | UUU | 926 | 1.27 | Ser | UCU | 563 | 1.68 |
|  |  | UUC | 528 | 0.73 |  | UCC | 342 | 1.02 |
|  | Leu | UUA | 829 | 1.82 |  | UCA | 415 | 1.24 |
|  |  | UUG | 579 | 1.27 |  | UCG | 185 | 0.55 |
|  |  | CUU | 564 | 1.24 | Pro | CCU | 427 | 1.51 |
|  |  | CUC | 195 | 0.43 |  | CCC | 219 | 0.78 |
|  |  | CUA | 376 | 0.83 |  | CCA | 333 | 1.18 |
|  |  | CUG | 189 | 0.42 |  | CCG | 149 | 0.53 |
|  | Ile | AUU | 1072 | 1.44 | Thr | ACU | 529 | 1.56 |
|  |  | AUC | 454 | 0.61 |  | ACC | 258 | 0.76 |
|  |  | AUA | 700 | 0.94 |  | ACA | 421 | 1.24 |
|  | Met | AUG | 630 | 1 |  | ACG | 151 | 0.44 |
|  | Val | GUU | 528 | 1.47 | Ala | GCU | 595 | 1.71 |
|  |  | GUC | 164 | 0.46 |  | GCC | 228 | 0.65 |
|  |  | GUA | 546 | 1.52 |  | GCA | 394 | 1.13 |
|  |  | GUG | 203 | 0.56 |  | GCG | 176 | 0.51 |
|  | Tyr | UAU | 765 | 1.59 | Cys | UGU | 225 | 1.47 |
|  |  | UAC | 200 | 0.41 |  | UGC | 81 | 0.53 |
|  | TER | UAA | 38 | 1.37 | Trp | UGG | 471 | 1 |
|  |  | UAG | 25 | 0.9 | Arg | CGU | 360 | 1.34 |
|  |  | UGA | 20 | 0.72 |  | CGC | 95 | 0.35 |
|  | His | CAU | 505 | 1.51 |  | CGA | 362 | 1.35 |
|  |  | CAC | 164 | 0.49 |  | CGG | 116 | 0.43 |
|  | Gln | CAA | 685 | 1.51 |  | AGA | 499 | 1.86 |
|  |  | CAG | 225 | 0.49 |  | AGG | 182 | 0.68 |
|  | Asn | AAU | 983 | 1.54 | Ser | AGU | 391 | 1.17 |
|  |  | AAC | 291 | 0.46 |  | AGC | 115 | 0.34 |
|  | Lys | AAA | 989 | 1.44 | Gly | GGU | 601 | 1.33 |
|  |  | AAG | 383 | 0.56 |  | GGC | 185 | 0.41 |
|  | Asp | GAU | 878 | 1.6 |  | GGA | 719 | 1.59 |
|  |  | GAC | 221 | 0.4 |  | GGG | 300 | 0.66 |
|  | Glu | GAA | 995 | 1.46 |  |  |  |  |
|  |  | GAG | 365 | 0.54 |  |  |  |  |
| *Aconitum episcopale* | Phe | UUU | 930 | 1.28 | Ser | UCU | 562 | 1.67 |
|  |  | UUC | 528 | 0.72 |  | UCC | 341 | 1.02 |
|  | Leu | UUA | 827 | 1.82 |  | UCA | 413 | 1.23 |
|  |  | UUG | 576 | 1.27 |  | UCG | 192 | 0.57 |
|  |  | CUU | 564 | 1.24 | Pro | CCU | 429 | 1.52 |
|  |  | CUC | 193 | 0.42 |  | CCC | 219 | 0.78 |
|  |  | CUA | 378 | 0.83 |  | CCA | 333 | 1.18 |
|  |  | CUG | 189 | 0.42 |  | CCG | 149 | 0.53 |
|  | Ile | AUU | 1074 | 1.45 | Thr | ACU | 530 | 1.55 |
|  |  | AUC | 457 | 0.62 |  | ACC | 257 | 0.75 |
|  |  | AUA | 697 | 0.94 |  | ACA | 428 | 1.25 |
|  | Met | AUG | 630 | 1 |  | ACG | 151 | 0.44 |
|  | Val | GUU | 527 | 1.47 | Ala | GCU | 594 | 1.71 |
|  |  | GUC | 162 | 0.45 |  | GCC | 228 | 0.66 |
|  |  | GUA | 547 | 1.52 |  | GCA | 393 | 1.13 |
|  |  | GUG | 202 | 0.56 |  | GCG | 177 | 0.51 |
|  | Tyr | UAU | 765 | 1.59 | Cys | UGU | 225 | 1.47 |
|  |  | UAC | 198 | 0.41 |  | UGC | 81 | 0.53 |
|  | TER | UAA | 38 | 1.37 | Trp | UGG | 470 | 1 |
|  |  | UAG | 25 | 0.9 | Arg | CGU | 358 | 1.33 |
|  |  | UGA | 20 | 0.72 |  | CGC | 94 | 0.35 |
|  | His | CAU | 509 | 1.51 |  | CGA | 363 | 1.35 |
|  |  | CAC | 164 | 0.49 |  | CGG | 118 | 0.44 |
|  | Gln | CAA | 684 | 1.51 |  | AGA | 499 | 1.86 |
|  |  | CAG | 222 | 0.49 |  | AGG | 181 | 0.67 |
|  | Asn | AAU | 991 | 1.55 | Ser | AGU | 392 | 1.17 |
|  |  | AAC | 290 | 0.45 |  | AGC | 114 | 0.34 |
|  | Lys | AAA | 990 | 1.44 | Gly | GGU | 601 | 1.33 |
|  |  | AAG | 387 | 0.56 |  | GGC | 185 | 0.41 |
|  | Asp | GAU | 881 | 1.6 |  | GGA | 722 | 1.59 |
|  |  | GAC | 222 | 0.4 |  | GGG | 304 | 0.67 |
|  | Glu | GAA | 996 | 1.46 |  |  |  |  |
|  |  | GAG | 366 | 0.54 |  |  |  |  |
| *Aconitum stapfianum* | Phe | UUU | 931 | 1.28 | Ser | UCU | 562 | 1.67 |
|  |  | UUC | 528 | 0.72 |  | UCC | 341 | 1.02 |
|  | Leu | UUA | 828 | 1.82 |  | UCA | 413 | 1.23 |
|  |  | UUG | 575 | 1.27 |  | UCG | 192 | 0.57 |
|  |  | CUU | 564 | 1.24 | Pro | CCU | 429 | 1.52 |
|  |  | CUC | 193 | 0.42 |  | CCC | 219 | 0.78 |
|  |  | CUA | 378 | 0.83 |  | CCA | 333 | 1.18 |
|  |  | CUG | 189 | 0.42 |  | CCG | 149 | 0.53 |
|  | Ile | AUU | 1074 | 1.45 | Thr | ACU | 530 | 1.55 |
|  |  | AUC | 457 | 0.62 |  | ACC | 257 | 0.75 |
|  |  | AUA | 697 | 0.94 |  | ACA | 428 | 1.25 |
|  | Met | AUG | 630 | 1 |  | ACG | 151 | 0.44 |
|  | Val | GUU | 527 | 1.47 | Ala | GCU | 594 | 1.71 |
|  |  | GUC | 162 | 0.45 |  | GCC | 228 | 0.66 |
|  |  | GUA | 547 | 1.52 |  | GCA | 393 | 1.13 |
|  |  | GUG | 202 | 0.56 |  | GCG | 177 | 0.51 |
|  | Tyr | UAU | 764 | 1.59 | Cys | UGU | 225 | 1.47 |
|  |  | UAC | 198 | 0.41 |  | UGC | 81 | 0.53 |
|  | TER | UAA | 38 | 1.37 | Trp | UGG | 470 | 1 |
|  |  | UAG | 25 | 0.9 | Arg | CGU | 358 | 1.33 |
|  |  | UGA | 20 | 0.72 |  | CGC | 94 | 0.35 |
|  | His | CAU | 509 | 1.51 |  | CGA | 363 | 1.35 |
|  |  | CAC | 164 | 0.49 |  | CGG | 118 | 0.44 |
|  | Gln | CAA | 684 | 1.51 |  | AGA | 499 | 1.86 |
|  |  | CAG | 222 | 0.49 |  | AGG | 181 | 0.67 |
|  | Asn | AAU | 991 | 1.55 | Ser | AGU | 392 | 1.17 |
|  |  | AAC | 290 | 0.45 |  | AGC | 114 | 0.34 |
|  | Lys | AAA | 990 | 1.44 | Gly | GGU | 601 | 1.33 |
|  |  | AAG | 387 | 0.56 |  | GGC | 185 | 0.41 |
|  | Asp | GAU | 881 | 1.6 |  | GGA | 722 | 1.59 |
|  |  | GAC | 222 | 0.4 |  | GGG | 304 | 0.67 |
|  | Glu | GAA | 996 | 1.46 |  |  |  |  |
|  |  | GAG | 366 | 0.54 |  |  |  |  |
| *Aconitum weixiense* | Phe | UUU | 931 | 1.28 | Ser | UCU | 562 | 1.67 |
|  |  | UUC | 527 | 0.72 |  | UCC | 341 | 1.02 |
|  | Leu | UUA | 827 | 1.82 |  | UCA | 413 | 1.23 |
|  |  | UUG | 576 | 1.27 |  | UCG | 192 | 0.57 |
|  |  | CUU | 564 | 1.24 | Pro | CCU | 428 | 1.52 |
|  |  | CUC | 193 | 0.42 |  | CCC | 220 | 0.78 |
|  |  | CUA | 378 | 0.83 |  | CCA | 333 | 1.18 |
|  |  | CUG | 189 | 0.42 |  | CCG | 149 | 0.53 |
|  | Ile | AUU | 1074 | 1.45 | Thr | ACU | 530 | 1.55 |
|  |  | AUC | 457 | 0.62 |  | ACC | 257 | 0.75 |
|  |  | AUA | 697 | 0.94 |  | ACA | 427 | 1.25 |
|  | Met | AUG | 631 | 1 |  | ACG | 150 | 0.44 |
|  | Val | GUU | 527 | 1.47 | Ala | GCU | 594 | 1.71 |
|  |  | GUC | 162 | 0.45 |  | GCC | 228 | 0.66 |
|  |  | GUA | 546 | 1.52 |  | GCA | 393 | 1.13 |
|  |  | GUG | 203 | 0.56 |  | GCG | 177 | 0.51 |
|  | Tyr | UAU | 766 | 1.59 | Cys | UGU | 225 | 1.47 |
|  |  | UAC | 198 | 0.41 |  | UGC | 81 | 0.53 |
|  | TER | UAA | 38 | 1.37 | Trp | UGG | 470 | 1 |
|  |  | UAG | 25 | 0.9 | Arg | AGA | 499 | 1.85 |
|  |  | UGA | 20 | 0.72 |  | AGG | 182 | 0.68 |
|  | His | CAU | 506 | 1.51 |  | CGU | 360 | 1.34 |
|  |  | CAC | 164 | 0.49 |  | CGC | 95 | 0.35 |
|  | Gln | CAA | 684 | 1.51 |  | CGA | 362 | 1.34 |
|  |  | CAG | 222 | 0.49 |  | CGG | 118 | 0.44 |
|  | Asn | AAU | 991 | 1.55 | Ser | AGU | 392 | 1.17 |
|  |  | AAC | 290 | 0.45 |  | AGC | 114 | 0.34 |
|  | Lys | AAA | 988 | 1.44 | Gly | GGU | 601 | 1.33 |
|  |  | AAG | 387 | 0.56 |  | GGC | 185 | 0.41 |
|  | Asp | GAU | 880 | 1.6 |  | GGA | 721 | 1.59 |
|  |  | GAC | 222 | 0.4 |  | GGG | 303 | 0.67 |
|  | Glu | GAA | 996 | 1.46 |  |  |  |  |
|  |  | GAG | 365 | 0.54 |  |  |  |  |
| *Aconitum nagarum* | Phe | UUU | 932 | 1.27 | Ser | UCU | 562 | 1.67 |
|  |  | UUC | 532 | 0.73 |  | UCC | 342 | 1.02 |
|  | Leu | UUA | 827 | 1.82 |  | UCA | 414 | 1.23 |
|  |  | UUG | 576 | 1.27 |  | UCG | 191 | 0.57 |
|  |  | CUU | 566 | 1.24 | Pro | CCU | 427 | 1.51 |
|  |  | CUC | 195 | 0.43 |  | CCC | 219 | 0.78 |
|  |  | CUA | 381 | 0.84 |  | CCA | 333 | 1.18 |
|  |  | CUG | 187 | 0.41 |  | CCG | 150 | 0.53 |
|  | Ile | AUU | 1074 | 1.45 | Thr | ACU | 529 | 1.55 |
|  |  | AUC | 454 | 0.61 |  | ACC | 257 | 0.75 |
|  |  | AUA | 696 | 0.94 |  | ACA | 426 | 1.25 |
|  | Met | AUG | 629 | 1 |  | ACG | 151 | 0.44 |
|  | Val | GUU | 529 | 1.47 | Ala | GCU | 592 | 1.7 |
|  |  | GUC | 165 | 0.46 |  | GCC | 229 | 0.66 |
|  |  | GUA | 544 | 1.51 |  | GCA | 394 | 1.13 |
|  |  | GUG | 203 | 0.56 |  | GCG | 177 | 0.51 |
|  | Tyr | UAU | 764 | 1.59 | Cys | UGU | 225 | 1.47 |
|  |  | UAC | 200 | 0.41 |  | UGC | 81 | 0.53 |
|  | TER | UAA | 38 | 1.37 | Trp | UGG | 471 | 1 |
|  |  | UAG | 25 | 0.9 | Arg | AGA | 498 | 1.85 |
|  |  | UGA | 20 | 0.72 |  | AGG | 181 | 0.67 |
|  | His | CAU | 507 | 1.51 |  | CGU | 359 | 1.34 |
|  |  | CAC | 163 | 0.49 |  | CGC | 95 | 0.35 |
|  | Gln | CAA | 686 | 1.51 |  | CGA | 363 | 1.35 |
|  |  | CAG | 225 | 0.49 |  | CGG | 117 | 0.44 |
|  | Asn | AAU | 988 | 1.55 | Ser | AGU | 392 | 1.17 |
|  |  | AAC | 290 | 0.45 |  | AGC | 114 | 0.34 |
|  | Lys | AAA | 988 | 1.44 | Gly | GGU | 602 | 1.33 |
|  |  | AAG | 385 | 0.56 |  | GGC | 185 | 0.41 |
|  | Asp | GAU | 881 | 1.60 |  | GGA | 717 | 1.59 |
|  |  | GAC | 220 | 0.40 |  | GGG | 301 | 0.67 |
|  | Glu | GAA | 998 | 1.46 |  |  |  |  |
|  |  | GAG | 366 | 0.54 |  |  |  |  |
| *Aconitum duclouxii* | Phe | UUU | 929 | 1.27 | Ser | UCU | 563 | 1.68 |
|  |  | UUC | 532 | 0.73 |  | UCC | 340 | 1.01 |
|  | Leu | UUA | 829 | 1.82 |  | UCA | 412 | 1.23 |
|  |  | UUG | 574 | 1.26 |  | UCG | 191 | 0.57 |
|  |  | CUU | 566 | 1.24 | Pro | CCU | 428 | 1.52 |
|  |  | CUC | 195 | 0.43 |  | CCC | 218 | 0.77 |
|  |  | CUA | 378 | 0.83 |  | CCA | 331 | 1.17 |
|  |  | CUG | 188 | 0.41 |  | CCG | 150 | 0.53 |
|  | Ile | AUU | 1074 | 1.45 | Thr | ACU | 530 | 1.56 |
|  |  | AUC | 454 | 0.61 |  | ACC | 256 | 0.75 |
|  |  | AUA | 694 | 0.94 |  | ACA | 424 | 1.25 |
|  | Met | AUG | 627 | 1.00 |  | ACG | 150 | 0.44 |
|  | Val | GUU | 530 | 1.47 | Ala | GCU | 592 | 1.7 |
|  |  | GUC | 164 | 0.45 |  | GCC | 230 | 0.66 |
|  |  | GUA | 545 | 1.51 |  | GCA | 394 | 1.13 |
|  |  | GUG | 203 | 0.56 |  | GCG | 177 | 0.51 |
|  | Tyr | UAU | 761 | 1.59 | Cys | UGU | 225 | 1.48 |
|  |  | UAC | 199 | 0.41 |  | UGC | 80 | 0.52 |
|  | TER | UAA | 38 | 1.37 | Trp | UGG | 471 | 1 |
|  |  | UAG | 25 | 0.9 | Arg | AGA | 498 | 1.85 |
|  |  | UGA | 20 | 0.72 |  | AGG | 183 | 0.68 |
|  | His | CAU | 506 | 1.51 |  | CGU | 359 | 1.34 |
|  |  | CAC | 163 | 0.49 |  | CGC | 95 | 0.35 |
|  | Gln | CAA | 682 | 1.5 |  | CGA | 362 | 1.35 |
|  |  | CAG | 226 | 0.5 |  | CGG | 116 | 0.43 |
|  | Asn | AAU | 986 | 1.54 | Ser | AGU | 390 | 1.16 |
|  |  | AAC | 292 | 0.46 |  | AGC | 114 | 0.34 |
|  | Lys | AAA | 986 | 1.44 | Gly | GGU | 602 | 1.33 |
|  |  | AAG | 385 | 0.56 |  | GGC | 185 | 0.41 |
|  | Asp | GAU | 880 | 1.6 |  | GGA | 716 | 1.59 |
|  |  | GAC | 220 | 0.4 |  | GGG | 301 | 0.67 |
|  | Glu | GAA | 997 | 1.46 |  |  |  |  |
|  |  | GAG | 366 | 0.54 |  |  |  |  |
| *Aconitum ouvrardianum* | Phe | UUU | 915 | 1.28 | Ser | UCU | 559 | 1.69 |
|  |  | UUC | 518 | 0.72 |  | UCC | 338 | 1.02 |
|  | Leu | UUA | 820 | 1.82 |  | UCA | 406 | 1.22 |
|  |  | UUG | 574 | 1.28 |  | UCG | 188 | 0.57 |
|  |  | CUU | 557 | 1.24 | Pro | CCU | 423 | 1.5 |
|  |  | CUC | 186 | 0.41 |  | CCC | 222 | 0.79 |
|  |  | CUA | 374 | 0.83 |  | CCA | 336 | 1.19 |
|  |  | CUG | 190 | 0.42 |  | CCG | 148 | 0.52 |
|  | Ile | AUU | 1069 | 1.45 | Thr | ACU | 526 | 1.56 |
|  |  | AUC | 455 | 0.62 |  | ACC | 255 | 0.76 |
|  |  | AUA | 692 | 0.94 |  | ACA | 419 | 1.24 |
|  | Met | AUG | 624 | 1 |  | ACG | 150 | 0.44 |
|  | Val | GUU | 525 | 1.47 | Ala | GCU | 596 | 1.71 |
|  |  | GUC | 158 | 0.44 |  | GCC | 229 | 0.66 |
|  |  | GUA | 541 | 1.52 |  | GCA | 393 | 1.13 |
|  |  | GUG | 203 | 0.57 |  | GCG | 175 | 0.5 |
|  | Tyr | UAU | 752 | 1.58 | Cys | UGU | 224 | 1.48 |
|  |  | UAC | 198 | 0.42 |  | UGC | 78 | 0.52 |
|  | TER | UAA | 38 | 1.34 | Trp | UGG | 475 | 1 |
|  |  | UAG | 25 | 0.88 | Arg | CGU | 356 | 1.34 |
|  | TER | UGA | 22 | 0.78 |  | CGC | 95 | 0.36 |
|  | His | CAU | 508 | 1.52 |  | CGA | 357 | 1.34 |
|  |  | CAC | 162 | 0.48 |  | CGG | 116 | 0.44 |
|  | Gln | CAA | 682 | 1.51 | Ser | AGU | 387 | 1.17 |
|  |  | CAG | 220 | 0.49 |  | AGC | 112 | 0.34 |
|  | Asn | AAU | 982 | 1.54 | Arg | AGA | 489 | 1.84 |
|  |  | AAC | 290 | 0.46 |  | AGG | 182 | 0.68 |
|  | Lys | AAA | 983 | 1.45 | Gly | GGU | 597 | 1.33 |
|  |  | AAG | 375 | 0.55 |  | GGC | 186 | 0.41 |
|  | Asp | GAU | 873 | 1.6 |  | GGA | 716 | 1.59 |
|  |  | GAC | 219 | 0.4 |  | GGG | 303 | 0.67 |
|  | Glu | GAA | 983 | 1.46 |  |  |  |  |
|  |  | GAG | 359 | 0.54 |  |  |  |  |
| *Aconitum delavayi* | Phe | UUU | 915 | 1.28 | Ser | UCU | 560 | 1.69 |
|  |  | UUC | 518 | 0.72 |  | UCC | 338 | 1.02 |
|  | Leu | UUA | 820 | 1.82 |  | UCA | 406 | 1.22 |
|  |  | UUG | 574 | 1.27 |  | UCG | 188 | 0.57 |
|  |  | CUU | 557 | 1.24 | Pro | CCU | 423 | 1.5 |
|  |  | CUC | 186 | 0.41 |  | CCC | 222 | 0.79 |
|  |  | CUA | 375 | 0.83 |  | CCA | 336 | 1.19 |
|  |  | CUG | 190 | 0.42 |  | CCG | 148 | 0.52 |
|  | Ile | AUU | 1069 | 1.45 | Thr | ACU | 527 | 1.56 |
|  |  | AUC | 455 | 0.62 |  | ACC | 255 | 0.76 |
|  |  | AUA | 691 | 0.94 |  | ACA | 415 | 1.23 |
|  | Met | AUG | 624 | 1 |  | ACG | 150 | 0.45 |
|  | Val | GUU | 525 | 1.47 | Ala | GCU | 595 | 1.71 |
|  |  | GUC | 158 | 0.44 |  | GCC | 229 | 0.66 |
|  |  | GUA | 541 | 1.52 |  | GCA | 393 | 1.13 |
|  |  | GUG | 203 | 0.57 |  | GCG | 175 | 0.5 |
|  | Tyr | UAU | 751 | 1.58 | Cys | UGU | 224 | 1.48 |
|  |  | UAC | 198 | 0.42 |  | UGC | 78 | 0.52 |
|  | TER | UAA | 38 | 1.34 | Trp | UGG | 475 | 1 |
|  |  | UAG | 25 | 0.88 | Arg | AGA | 489 | 1.84 |
|  |  | UGA | 22 | 0.78 |  | AGG | 182 | 0.68 |
|  | His | CAU | 508 | 1.52 |  | CGU | 356 | 1.34 |
|  |  | CAC | 162 | 0.48 |  | CGC | 95 | 0.36 |
|  | Gln | CAA | 682 | 1.51 |  | CGA | 357 | 1.34 |
|  |  | CAG | 220 | 0.49 |  | CGG | 116 | 0.44 |
|  | Asn | AAU | 978 | 1.54 | Ser | AGU | 387 | 1.17 |
|  |  | AAC | 291 | 0.46 |  | AGC | 112 | 0.34 |
|  | Lys | AAA | 983 | 1.45 | Gly | GGU | 597 | 1.33 |
|  |  | AAG | 371 | 0.55 |  | GGC | 186 | 0.41 |
|  | Asp | GAU | 870 | 1.6 |  | GGA | 712 | 1.59 |
|  |  | GAC | 219 | 0.4 |  | GGG | 299 | 0.67 |
|  | Glu | GAA | 983 | 1.46 |  |  |  |  |
|  |  | GAG | 359 | 0.54 |  |  |  |  |
| *Aconitum ramulosum* | Phe | UUU | 915 | 1.28 | Ser | UCU | 560 | 1.69 |
|  |  | UUC | 518 | 0.72 |  | UCC | 338 | 1.02 |
|  | Leu | UUA | 820 | 1.82 |  | UCA | 406 | 1.22 |
|  |  | UUG | 574 | 1.27 |  | UCG | 188 | 0.57 |
|  |  | CUU | 557 | 1.24 | Pro | CCU | 423 | 1.5 |
|  |  | CUC | 186 | 0.41 |  | CCC | 222 | 0.79 |
|  |  | CUA | 375 | 0.83 |  | CCA | 336 | 1.19 |
|  |  | CUG | 190 | 0.42 |  | CCG | 148 | 0.52 |
|  | Ile | AUU | 1069 | 1.45 | Thr | ACU | 527 | 1.56 |
|  |  | AUC | 455 | 0.62 |  | ACC | 255 | 0.75 |
|  |  | AUA | 691 | 0.94 |  | ACA | 421 | 1.24 |
|  | Met | AUG | 624 | 1 |  | ACG | 150 | 0.44 |
|  | Val | GUU | 525 | 1.47 | Ala | GCU | 595 | 1.71 |
|  |  | GUC | 158 | 0.44 |  | GCC | 229 | 0.66 |
|  |  | GUA | 541 | 1.52 |  | GCA | 393 | 1.13 |
|  |  | GUG | 203 | 0.57 |  | GCG | 175 | 0.5 |
|  | Tyr | UAU | 751 | 1.58 | Cys | UGU | 224 | 1.48 |
|  |  | UAC | 198 | 0.42 |  | UGC | 78 | 0.52 |
|  | TER | UAA | 38 | 1.34 | Trp | UGG | 475 | 1 |
|  |  | UAG | 25 | 0.88 | Arg | CGU | 356 | 1.34 |
|  |  | UGA | 22 | 0.78 |  | CGC | 95 | 0.36 |
|  | His | CAU | 508 | 1.52 |  | CGA | 357 | 1.34 |
|  |  | CAC | 162 | 0.48 |  | CGG | 116 | 0.44 |
|  | Gln | CAA | 682 | 1.51 | Ser | AGU | 387 | 1.17 |
|  |  | CAG | 220 | 0.49 |  | AGC | 112 | 0.34 |
|  | Asn | AAU | 984 | 1.54 | Arg | AGA | 489 | 1.84 |
|  |  | AAC | 291 | 0.46 |  | AGG | 182 | 0.68 |
|  | Lys | AAA | 983 | 1.45 | Gly | GGU | 597 | 1.32 |
|  |  | AAG | 377 | 0.55 |  | GGC | 186 | 0.41 |
|  | Asp | GAU | 876 | 1.6 |  | GGA | 718 | 1.59 |
|  |  | GAC | 219 | 0.4 |  | GGG | 305 | 0.68 |
|  | Glu | GAA | 983 | 1.46 |  |  |  |  |
|  |  | GAG | 359 | 0.54 |  |  |  |  |

**Table S5.** SSR motifs in ten *Aconitum* chloroplast genomes.

| **SSR**  **Type** | **Repeat**  **Unit** | ***A. vilmorinianum*** | ***A. stylosum*** | ***A. episcopale*** | ***A. stapfianum*** | ***A. weixiense*** | ***A. nagarum*** | ***A. duclouxii*** | ***A. ouvrardianumi*** | ***A. delavayii*** | ***A. ramulosum*** | **Total** |
| --- | --- | --- | --- | --- | --- | --- | --- | --- | --- | --- | --- | --- |
| Mono- | A/T | 35 | 33 | 34 | 31 | 36 | 30 | 29 | 31 | 29 | 29 | 317 |
|  | G/C | 1 |  |  | 1 |  |  |  | 1 | 1 | 1 | 5 |
| Di- | AT/AT | 15 | 16 | 15 | 15 | 16 | 16 | 16 | 16 | 16 | 16 | 157 |
| Tri- | AAT/ATT | 5 | 6 | 5 | 5 | 5 | 6 | 5 | 3 | 5 | 5 | 62 |
|  | ATC/ATG | 1 | 1 | 1 | 1 | 1 | 1 | 1 | 1 | 1 | 1 |  |
|  | CCG/CGG |  |  |  |  |  | 1 | 1 |  |  |  |  |
| Tetra- | AAAG/CTTT | 3 | 3 | 3 | 3 | 3 | 3 | 3 | 3 | 3 | 3 | 71 |
|  | AAAT/ATTT | 4 | 2 | 4 | 4 | 4 | 2 | 2 | 3 | 3 | 3 |  |
|  | AATG/ATTC | 1 | 1 | 1 | 1 | 1 | 1 | 1 | 1 | 1 | 1 |  |
| Penta- | AAAAT/ATTTT | 2 |  |  |  | 2 |  |  |  |  |  | 21 |
|  | AAATT/AATTT | 1 |  | 1 |  | 1 |  |  | 1 |  |  |  |
|  | AATAT/ATATT | 1 | 1 | 1 | 1 | 1 | 1 | 1 | 2 | 1 | 1 |  |
|  | ACTAT/AGTAT |  |  |  |  |  | 1 | 1 |  |  |  |  |
| Hexa- | AACTCT/AGAGTT |  |  |  | 1 |  |  |  |  | 1 | 1 | 4 |
|  | AATTAT/AATTAT |  | 1 |  |  |  |  |  |  |  |  |  |
| Total |  | 69 | 64 | 65 | 63 | 70 | 62 | 60 | 62 | 61 | 61 | 637 |

**Table S6.** Chloroplast genome information table for 44 species in the phylogenetic tree.

| **GenBank acc.** | **Species** | **Plastome size (bp)** | **genus** |
| --- | --- | --- | --- |
| NC_036357.1 | *Aconitum angustius* W. T. Wang | 156,109 | *Aconitum* |
| NC_036358.1 | *Aconitum finetianum* Hand.-Mazz. | 155,625 | *Aconitum* |
| NC_036359.1 | *Aconitum sinomontanum* Nakai | 157,215 | *Aconitum* |
| MK253470.1 | *Aconitum barbatum* Pers. | 156,761 | *Aconitum* |
| KT820664.1 | *Aconitum barbatum* var. *hispidum* (DC.) Seringe | 156,782 | *Aconitum* |
| MN967020.1 | *Aconitum puchonroenicum* Uyeki & Sakata | 155,631 | *Aconitum* |
| NC_057536.1 | *Aconitum quelpaertense* Nakai | 155,636 | *Aconitum* |
| MW817090.1 | *Aconitum scaposum* Franch*.* | 157,688 | *Aconitum* |
| MW246162.1 | *Aconitum scaposum* var. *Vaginatum* (Pritz.) Rapaics | 157,493 | *Aconitum* |
| MF186593.1 | *Aconitum reclinatum* A.Gray | 157,354 | *Aconitum* |
| NC_035894.1 | *Aconitum longecassidatum* Nakai | 155,524 | *Aconitum* |
| NC_035892.1 | *Aconitum pseudolaeve* Nakai | 155,628 | *Aconitum* |
| NC_031410.1 | *Aconitum austrokoreense* Koidz. | 155,682 | *Aconitum* |
| NC_029829.1 | *Aconitum chiisanense* Nakai | 155,934 | *Aconitum* |
| NC_031420.1 | *Aconitum ciliare* DC. | 155,832 | *Aconitum* |
| NC_031422.1 | *Aconitum kusnezoffii* Reichb. | 155,862 | *Aconitum* |
| KT820668.1 | *Aconitum jaluense* subsp*. jaluense* | 155,884 | *Aconitum* |
| KT820670.1 | *Aconitum japonicum* subsp. *napiforme* Thunberg | 155,878 | *Aconitum* |
| NC_031423.1 | *Aconitum monanthum* Nakai | 155,688 | *Aconitum* |
| NC_030761.1 | *Aconitum carmichaelii* Debeaux | 155,737 | *Aconitum* |
| KU556690.1 | *Aconitum volubile* Pall. ex Koeue | 155,872 | *Aconitum* |
| NC_038098.1 | *Aconitum contortum* Finet et Gagnep. | 155,653 | *Aconitum* |
| NC_038095.1 | *Aconitum hemsleyanum* Pritz. | 155,684 | *Aconitum* |
| MZ169393.1 | *Aconitum piepunense* Hand.-Mazz. | 155,836 | *Aconitum* |
| NC_053848.1 | *Aconitum pendulum* Busch. | 155,597 | *Aconitum* |
| NC_031421.1 | *Aconitum coreanum* (Lévl.) Rapaics | 157,029 | *Aconitum* |
| KC844054.1 | *Aconitum barbatum* var. *puberulum* Ledeb*.* | 156,749 | *Aconitum* |
| NC_041579.1 | *Aconitum brachypodum* Diels. | 155,651 | *Aconitum* |
| NC_056280.1 | *Aconitum flavum* Hand.-Mazz. | 155,654 | *Aconitum* |
| NC_050689.1 | *Aconitum tanguticum* (Maxim.) Stapf | 157,114 | *Aconitum* |
| OM328065.1 | *Aconitum vilmorinianum* Kom. | 155,921 | *Aconitum* |
| OM328071.1 | *Aconitum stylosum* Stapf | 155,475 | *Aconitum* |
| OM328066.1 | *Aconitum episcopale* Leveille | 155,853 | *Aconitum* |
| OM328067.1 | *Aconitum stapfianum* Hand.-Mazz. | 155,858 | *Aconitum* |
| OM328069.1 | *Aconitum weixiense* W. T. Wang | 155,872 | *Aconitum* |
| OM328068.1 | *Aconitum nagarum* Stapf | 155,732 | *Aconitum* |
| OM328070.1 | *Aconitum duclouxii* Lévl*.* | 155,479 | *Aconitum* |
| OM289057.1 | *Aconitum ouvrardianum* Hand.-Mazz. | 155,799 | *Aconitum* |
| OM289058.1 | *Aconitum delavayi* Franch. | 155,733 | *Aconitum* |
| OM289059.1 | *Aconitum ramulosum* W. T. Wang | 155,841 | *Aconitum* |
| OK323949.1 | *Aconitum bulleyanum* | 155,791 | *Aconitum* |
| OK539525.1 | *Aconitum habaense* | 155,800 | *Aconitum* |
| MK253461.1 | *Delphinium anthriscifolium* Hance | 155,077 | *Delphinium* |
| NC_049872.1 | *Delphinium grandiflorum* L. | 157,339 | *Delphinium* |


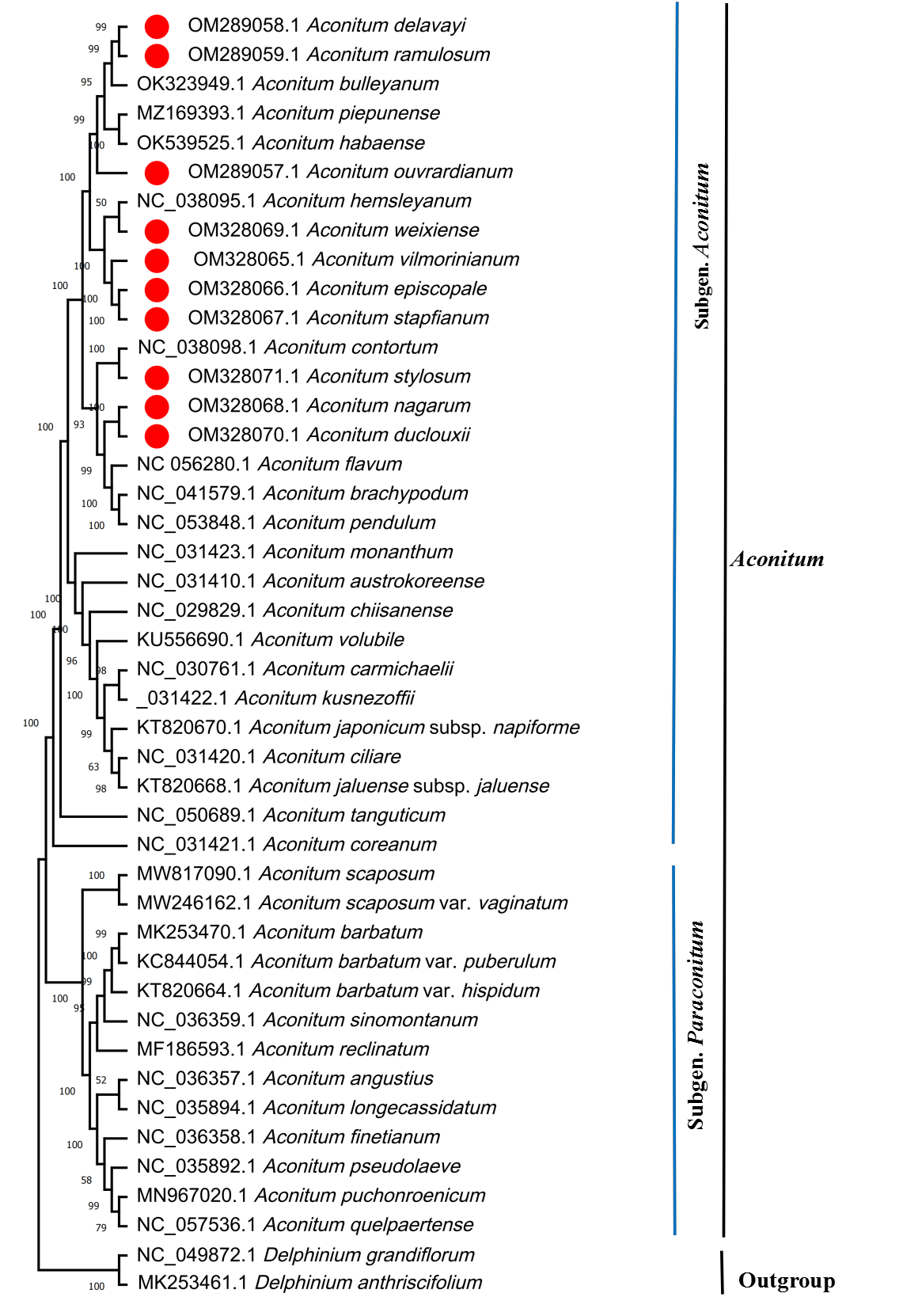


**Figure S1** The neighbor joining (NJ) method was used to construct a phylogenetic tree based on the 42 chloroplast genomes of of the genus *Aconitum.*
